# Selective Narrowing of the Bonding Modes of Plasmonic Nanoantennas

**DOI:** 10.1021/acsami.6c05142

**Published:** 2026-06-02

**Authors:** Ora Bitton, Lothar Houben, Hagai Cohen, Katya Rechav, Sigal Keshet, Anna Kossoy, Marek Patočka, Eran Mishuk, Bar Cohn, Lev Chuntonov, Alexander Vaskevich, Gilad Haran

**Affiliations:** † Department of Chemical Research Support, Faculty of Chemistry, 34976 Weizmann Institute of Science, Rehovot 7610001, Israel; ‡ Schulich Faculty of Chemistry, Technion−Israel Institute of Technology, Haifa 3200003, Israel; § Solid State Institute, Technion−Israel Institute of Technology, Haifa 3200003, Israel; ∥ The Helen Diller Quantum Center, Technion−Israel Institute of Technology, Haifa 3200003, Israel; ⊥ Department of Chemical and Biological Physics, Faculty of Chemistry, 34976 Weizmann Institute of Science, Rehovot 7610001, Israel

**Keywords:** plasmonic nanoantennas, localized surface plasmon, quality factor, line width narrowing, longitudinal plasmon mode, mode selectivity, strong coupling

## Abstract

Plasmonic bowtie-structured nanocavities are valuable structures for applications due to the large confinement of light at their center and the associated large enhancement of the electric field. Plasmonic bowties suffer, though, from broad spectral lines that limit their interaction with quantum emitters. We report here a significant narrowing of the longitudinal plasmon resonance that couples the two prisms of silver bowties, induced by the deposition of a thin (1 nm) Ti adhesion layer during fabrication. The quality factor of the longitudinal mode of many of these bowties is much larger than that of bowties without an adhesion layer, even while their transverse mode remains unmodified. Cross-sectional electron microscopy analysis reveals that the Ti adhesion layer is fully oxidized. Further, and surprisingly, the TiO_2_ adhesion layer is found to cause the bowties to partially embed in the silica substrate, thereby increasing the effective dielectric coefficient of their environment. Analysis of the results shows that the increase in the quality factor might be partially attributed to a reduction in radiative damping. The ability to obtain plasmon excitations of bowties with large quality factors is particularly attractive for multiple applications involving optical coupling to quantum emitters.

## Graphical abstract

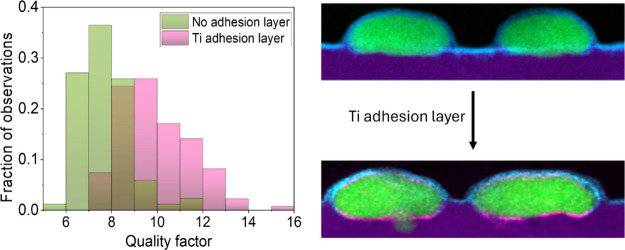

## 1 Introduction

Localized surface plasmons (LSPs) are quantized collective oscillations of conduction electrons sustained by metal nanoparticles.^1–3^ The electromagnetic fields associated with LSPs are strongly confined to their surfaces and enhanced. The outstanding ability of plasmonic nanostructures to confine light within volumes much smaller than that of the wavelength of light makes them one of the most promising platforms for various nanoscale photonic applications, including surface-enhanced Raman spectroscopy (SERS),^4^ surface enhanced fluorescence,^5^ molecular sensing^6^ and enhanced light-matter interactions both in the weak and strong coupling regimes.^7–10^

Near-field coupling between individual plasmonic nanoparticles leads to the formation of regions with highly localized and intense electromagnetic fields within the gaps of coupled structures, which are effectively turned into optical cavities (usually called plasmonic cavities, PCs).^7,11,12^ Microscopically, near-field coupling results in hybridization of the LSP modes of the individual nanoparticle, with the appearance of bright bonding modes and dark antibonding modes.^13,14^ The plasmonic bowtie (BT) cavity, comprised of two closely spaced metallic prisms, is found to be a most favorable structure for various applications due to extreme light confinement and electric field enhancement in the BT gap and due to the rich set of modes it possesses.^10,15–19^

The optical response and decay dynamics in PCs are governed by the two fundamental decay processes: radiative damping, in which energy is emitted as photons into the far field, and nonradiative damping, in which energy is dissipated within the metal or at the surface through processes such as ohmic losses, Landau damping, and chemical interface interactions.^1,20,21^ The total damping rate, γ,(the sum of radiative and nonradiative contributions) of an LSP corresponds to the line width (Δω) of its scattering spectral peak. A quality factor, *Q*, is introduced based on this definition as *Q* = ω_0_/Δω, where ω_0_ is the LSP resonance frequency. Both decay processes contribute to the total damping rate and are strongly influenced by effects such as particle geometry, mode symmetry, near-field coupling strength and environmental conditions.

While PCs are able to focus the electromagnetic field into deep subwavelength volumes, they are characterized by large γ, due to the lossy nature of metals. Accordingly, the typical *Q* values of PCs are rather moderate *Q* (∼5–20), far below those of dielectric cavities (∼ 10^3^–10^8^).^7,22^ However, since their mode volumes, *V*, are exceedingly small, their figures of merit, $\frac{Q}{V}$ , are large, making them attractive for a range of light-matter applications.

Much work has been performed on the impact of various factors on the γ of single metallic particles such as nanospheres, nanorods and nanodiscs.^23–33^ A few examples of such factors include size,^24,27–30^ material,^27,29,30^ geometric structure of the nanoparticles,^23–25,27^ their surface chemical composition^31–33^ and optical properties of the surrounding media.^24,25,29^ Recently, studies have focused on the impact of the properties of the adhesion layer —the layer deposited beneath the metallic nanostructures during fabrication to ensure good adhesion of metal and substrate—on LSP damping. Various studies demonstrated broadening of LSP spectral line width of individual nanoparticles in the presence of titanium (Ti) and chromium (Cr) adhesion layers and attributed this effect to the dissipative dielectric function of the metallic layer^34–38^ and in some cases also to chemical interface damping.^36^ In this context, metallic adhesion layers primarily increase nonradiative damping by introducing additional absorption pathways and enhancing interfacial electronic scattering. Studies on plasmonic gap modes probed the impact of both metallic layers and dielectric adhesion layers such as TiO_2_ and Cr_2_O_3_ on the plasmonic enhancement of single-molecule fluorescence and on the near-field damping.^34,37^ They show that SERS intensity and fluorescence enhancement with a TiO_2_ adhesion layer can be two times^37^ and 3.5 times^34^ larger, respectively, than with a Ti metallic layer. Therefore, one way to reduce damping is to use less absorptive adhesion materials such as Cr_2_O_3_, ITO, and specifically TiO_2_, which is characterized by negligible absorption.^34,37^

Experiments on the *Q* of ***coupled*** structures of plasmonic particles such as nanoparticle dimers^39–44^ and BT structures have been performed as well.^45–47^ For instance, Jiao et al. numerically modeled the near-field resonances of gold (Au) BTs in the presence of relatively thick adhesion layers. They found that the greatest field enhancement within the gap region is achieved either without an adhesion layer or with a TiO_2_ layer.^45^ In a previous work we show that by eliminating a Cr adhesion layer under PCs, we can significantly decrease the damping rate of both bright and dark LSP modes of silver BTs.^48^ Specifically, we demonstrate a significant increase in *Q*, leading to values of *Q*∼ 7.6–9.5, thereby enhancing their suitability for interaction with a single quantum dot (QD) in the strong coupling regime.^48^ In this work, we report a surprising finding: the line width of the coupled LSP mode of silver BTs can be narrowed by up to a factor of 2 by the addition of a thin Ti layer, while the line width of the transverse mode remains constant. We combine spectroscopy with chemical analysis and cross-sectional electron microscopy to elucidate the origin of this phenomenon. We show that when a seed layer of Ti is deposited (≤1 nm), it fully oxidizes, concomitantly leading to embedding of the BT structures into the underlying substrate and increasing their *Q*. By analyzing the scattering intensity, we find the line width narrowing in the longitudinal mode involves a decrease in the radiative decay rate. This contradicts the conventional expectations, where an increased refractive index typically enhances radiative damping.^49^ This mode-dependent response highlights the fundamentally different behavior of longitudinal and transverse plasmon modes in BT nanoantennas.

## 2 Results and Discussion

### 2.1 Design and Fabrication of Silver BT Cavities

We fabricate Ag BTs with a prism length of 80 nm by electron beam lithography on 18 nm thick silica (SiO_2_) membranes. By varying the electron-beam dose, we systematically modulate the BT gap from a few nanometers up to 40 nm, thereby tuning the localized surface plasmon (LSP) resonance energy. These dimensions were selected based on our previous systematic optimization of BT antennas for plasmon–exciton coupling with CdSe-based QDs, where a side length of approximately 80 nm yields a plasmon resonance near 1.8–2.0 eV, overlapping with the emission energies of the QDs, while remaining sufficiently large to preserve the BT geometry required for strong near-field confinement.^10,48^ Smaller antennas blue-shift the resonance toward higher energies compatible with available QDs, but at the cost of reduced tip sharpness, whereas larger structures redshift the plasmon resonance away from the available QD exciton energy. To enable correlative measurements on individual BT, the BTs were spaced 10 μm apart and uniquely marked. This layout allows the same BT to be identified and probed by optical scattering measurements and subsequently by transmission electron microscope (TEM) characterization. The BTs were fabricated following our previously established protocol.^10^ Briefly, electron beam lithography was used to pattern BTs in a PMMA resist layer. A Ti adhesion layer with a nominal thickness ranging from 0.1 to 5 nm was then deposited onto the patterned PMMA, followed by a 25 nm silver (Ag) layer. After PMMA lift-off, the remaining structures consist of Ag BTs on the substrate. To overcome the instability of Ag BTs at ambient environment and their high tendency to degrade,^50^ after resist lift-off we protect the structures by depositing a 5 nm alumina (Al_2_O_3_) layer on top of the structures, using atomic-layer deposition (ALD). For more details see Methods. A scanning electron microscope (SEM) image and a profile sketch of an Ag BT with a 1 nm Ti adhesion layer (BT_Ag–Ti_) are shown in Figure 1a top and bottom, respectively.

**Figure 1:**
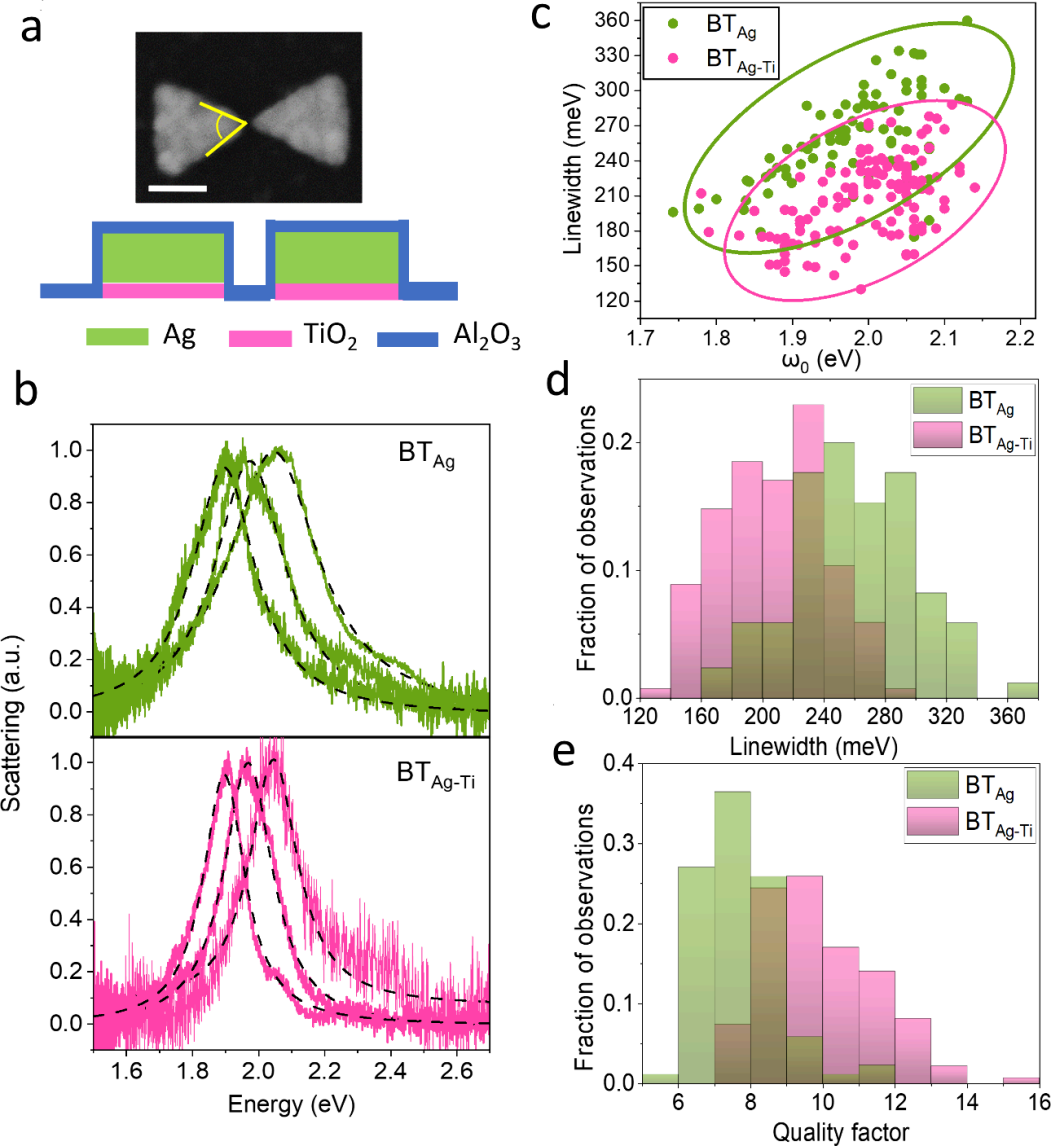
(a) Scanning electron microscope image (top) and a scheme (bottom) of a BT_Ag–Ti_ configuration. Scale bar is 50 nm and the tip angle (yellow) is ∼60°. (b) Scattering spectra of BT_Ag_ and BT_Ag–Ti_ with comparable LSP resonance energy of ω_0_ = 1.89, 1.96, and 2.04 eV. The black dashed lines correspond to Lorentzian fits, from which the line widths (fwhm) were obtained as 233, 260, and 333 meV for BT_Ag_ and 154, 180, and 177 meV for BT_Ag–Ti_. (c) LSP spectral line width values of BT_Ag_s and BT_Ag–Ti_s as a function of plasmon resonance frequency in energy units. For each group, the line width distributions were statistically compared using a two-sample Kolmogorov–Smirnov test, yielding statistically significant differences cases (*p* = 1.84 × 10^–14^), indicating that the distributions are unlikely to originate from the same underlying population. Pink and green curves are 2D confidence ellipses generated with the *2D Confidence Ellipse* tool in Origin, which fits an ellipse using the covariance matrix of the point coordinates. The centers of the green and pink ellipses in (c) are (1.974, 259.518) and (1.995, 205.99), respectively. (d, e) Histograms showing the distributions of plasmon spectral line widths and quality factors across the BT samples, plotted as fractions of observations rather than absolute counts. Mean and standard deviation values of all data points are given in Table S1 in the Supporting Information.

To characterize the Ti layers, we perform X-ray photoelectron spectroscopy (XPS) on BT structures with three different layer-by-layer configurations, as shown in Figure S1 in the Supporting Information. XPS spectra for a 1 nm layer of deposited Ti, whether at the bottom or top of the Ag BTs (Figures S1a and S1c, respectively), revealed a peak at 459.1 eV, corresponding to the TiO_2_ 2p_3/2_ band together with its accompanying peak at 465 eV, corresponding to the TiO_2_ 2p_1/2_. No Ti peak corresponding to the metallic-Ti band was detected (Figures S1a,c). In contrast, BTs with a 5 nm thick adhesion layer (Figure S1b), showed, together with the peak corresponding to TiO_2_, signals at lower binding energies, mainly at 453.9 eV. The latter corresponds to metallic Ti (2p_3/2_) and there are also fingerprints of intermediate, suboxide states. These results indicate that thin Ti layers fully oxidize, leading to pure TiO_2_ layers, while thicker layers remain partially metallic. We obtain similar results with samples in which flat continuous layers of Ti and Ag were deposited. Our results are in agreement with previous studies^51,52^ which show that an ultrathin layer of Ti is fully oxidized after evaporation.

### 2.2 Dark-Field Spectroscopy Demonstrates Narrowing of Longitudinal BT Modes

Light scattering from individual BTs was measured using a dark-field microscope with the polarization of the excitation light parallel to the BT axes. Figure 1b shows three scattering spectra of BTs without an adhesion layer (BT_Ag_, green curve) and three with a 1 nm Ti adhesion layer (BT_Ag–Ti_, pink curve), exhibiting comparable LSP resonance energies of ω_0_ ≈ 1.89 eV, 1.96 and 2.04 eV. Remarkably, the spectral line widths (Δω), extracted from Lorentzian fits to the spectra, are 233 meV, 260 and 333 meV for BT_Ag_ and 154 meV, 180 and 177 meV for BT_Ag–Ti_, indicating a significant narrowing of the scattering spectra upon the introduction of Ti. Figure 1c shows the spectral line widths obtained from a series of spectra without (BT_Ag_, green spots) and with (BT_Ag–Ti_, pink spots) 1 nm Ti adhesion layers as a function of ω_0_ (in energy units). Similar plot for the Quality factor is shown in Figure S2. These plots clearly demonstrate a significant decrease of Δω (Figure 1c) and a corresponding increase of *Q* (Figure S2) in the presence of the oxidized adhesion layer. We find that Δω can be as small as 140 meV and *Q* can reach ∼ 14, which is twice the lowest value of *Q*, obtained without an adhesion layer. These numbers are to be compared to *Q* ∼ 7.6–9.5 achieved in our earlier work on Ag BTs^48^ and *Q*∼ 4–9 estimated from spectra of Au BTs in other studies.^53–58^ Figures 1d,e show histograms of the spectral line width and quality factor distributions across the BT samples, where the bin heights represent the fraction of observations of BTs within specific line width and quality-factor ranges, respectively. The normalized histograms reveal a distinct subpopulation (∼10%) of BTs with exceptionally narrow line widths not observed for bare BTs, alongside a second subpopulation (50%) at line widths attainable for bare BTs but with much lower probability. Importantly, the line width narrowing effect is robust across different substrates: Δω values from each individual substrate, plotted in different colors in Figure S3, span the entire area of the fitted confidence ellipse, confirming that the trend is not substrate-specific.

We compare the scattering spectra obtained from the longitudinal plasmonic mode of BTs with LSP resonance energy of ω_0_ = 1.94 ± 0.02 eV to those of two additional plasmonic modes, namely the transverse mode of a BT (i.e., with the excitation light perpendicular to the BT axis) and that of a single prism at resonance energy ω_0_ = 2.05 ± 0.05 eV. Representative scattering spectra of prisms and transverse modes are shown in Figures S4a,b. The line width values obtained from these studies are shown in Figure 2, and a similar comparison of all BTs and prisms, characterized across a wide range of resonance energies, is presented in Figure S5. Clearly, the narrowing effect of the Ti layer is prominent in the case of the BT longitudinal mode, while for the transverse mode and for single prisms, this effect does not seem to exist.

**Figure 2:**
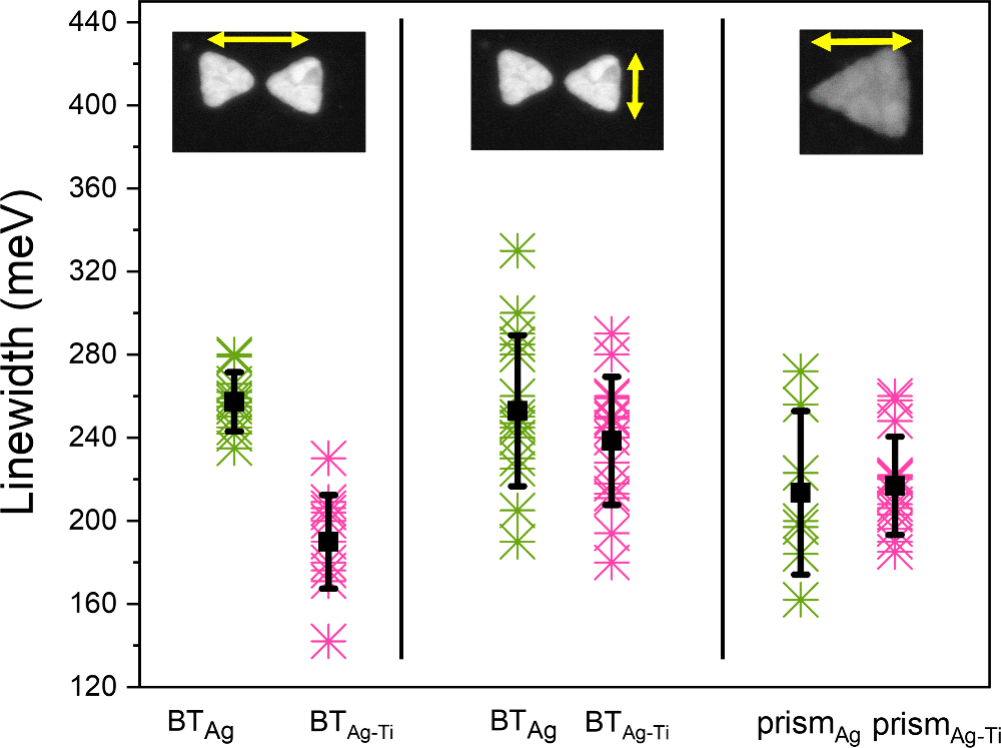
Spectral line widths of longitudinal modes of BT_Ag_s and BT_Ag–Ti_s with LSP resonance energy of 1.94 ± 0.02 eV (left), transverse modes of BT_Ag_s and BT_Ag–Ti_s (center), and prism_Ag_s and prism_Ag–Ti_s with LSP resonance energy of 2.05 ± 0.05 eV (right). Black squares and black error bars are the mean values and the standard deviations, respectively. Yellow arrows indicate the direction of light polarization. Mean and standard deviation values of all data points are shown in Table S1 in the Supporting Information.

We attempt to shed more light on the narrowing of plasmon line widths by further increasing the adhesion layer thickness from 1 to 5 nm. Interestingly, for a 5 nm Ti layer the line width increased dramatically and could reach up to 700 meV, as shown in Figure S6. The increase in the LSP line width for Ti thicknesses greater than 3 nm is not surprising and is consistent with the change in Ti phases present in the adhesion layer, as shown in Figure S1. For the thickness 5 nm the Ti is not fully oxidized (see Section 2.1 and Figure S1b) and the presence of a metallic state of Ti increases damping.^34,36,38,45^ However, the observation that a thin layer of dielectric material, TiO_2_, as obtained for the BT longitudinal mode in the case of a 1 nm deposited Ti, yields a significant decrease of the LSP line width is surprising.

We fabricate BTs with two additional configurations, one in which Ti was deposited both at the bottom and on top of the silver structures (BT_Ti–Ag–Ti_), and a second in which Ti was deposited just on top of the silver (BT_Ti–Ag_). In both configurations, the Ti and Ag depositions were carried out in sequence without exposing the sample to air. Importantly, the Ti layer deposited on top of the Ag layer was found by XPS to be fully oxidized, similarly to the bottom Ti layer (see Figure S1c). We measure scattering spectra for three cases: BT_Ti–Ag–Ti_ with bottom and top Ti layers of 0.2 and 1 nm, respectively, and BT_Ti–Ag_ with a top Ti layer of 1 nm. Representative scattering spectra of these configurations are shown in Figures S4c–e. A comparison of the spectral line widths of these structures is shown in Figure 3, which reveals that Ti deposition only on top of the BTs does not narrow the spectral line width, while deposition on both bottom and top of the BTs leads to narrower spectra, similar to the case of a Ti layer at the bottom only. This indicates that it is the bottom (oxidized) Ti layer that is responsible for the decrease of LSP line width.

**Figure 3:**
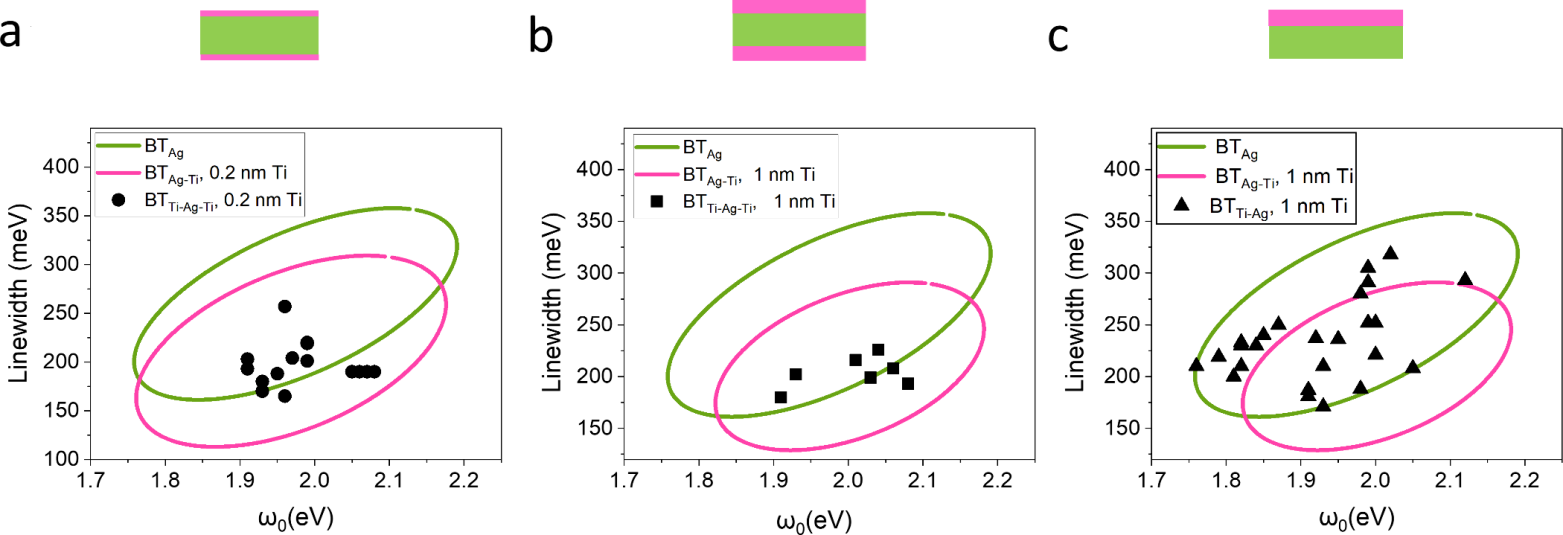
(a) LSP spectral line widths as a function of plasmon resonance frequency in energy units of (a) BT_Ti–Ag–Ti_s with nominal 0.2 nm deposited Ti (black spots), together with ellipse fits to BT_Ag_s and BT_Ag–Ti_s with nominal 0.2 nm deposited Ti. (b) BT_Ti–Ag–Ti_s with nominal 1 nm deposited Ti (black squares), together with ellipse fits to BT_Ag_s and BT_Ag–Ti_s with 1 nm deposited Ti. (c) BT_Ti–Ag_s with nominal 1 nm deposited Ti (black triangles), together with ellipse fits to BT_Ag_s and BT_Ag–Ti_s with nominal 1 nm deposited Ti. Top schemes correspond to the BT configuration. Mean and standard deviation values of all data points are shown in Table S1 in the Supporting Information.

### 2.3 Differences in Crystallinity Do Not Explain Variations in Line Width

In an attempt to understand this phenomenology, we first examine the crystalline structure of deposited silver directly on SiO_2_ substrate and on thin TiO_2_ layer by X-ray diffraction (XRD) measurement. Smaller grain sizes imply a larger density of grain boundaries, which should in principle enhance damping.^59,60^ Figure S7 shows the in-plane diffraction pattern of a film with and without a TiO_2_ layer at the interface. Peak analysis reveals that crystals are randomly oriented, with four typical directions present. The average grain size of a silver film deposited on TiO_2_ is estimated to be smaller by ∼ 20% than that of a silver film deposited directly on SiO_2_. This smaller grain size obtained for silver deposited on TiO_2_ cannot explain the observed decreased damping. We further test whether the smaller grain sizes of BT_Ti–Ag_ compared to BT_Ag_ leads to smoother films, which might lead to decreased damping. Atomic Force Microscope (AFM) scans of several BTs with and without deposited Ti yield RMS roughness values of 6.31 and 5.4 nm for BT_Ti–Ag_ and BT_Ag_, respectively. Therefore, roughness also does not seem to be the origin of LSP spectral line width narrowing.

### 2.4 Elemental Mapping of BT Cross Sections Reveals Embedding in Silica Surface

We use a focused ion beam (FIB) microscope to prepare cross-sectional lamellas of two types of BTs, namely BT_Ag_ and BT_Ti–Ag–Ti_, the latter with a Ti layer of 0.2 nm. Figure S8a displays high-resolution scanning electron transmission microscope (STEM) bright field images of the cross-sectional lamellas. Scattering spectra from these samples are shown in Figure S8b, demonstrating spectral line width of 343 and 168 meV for BT_Ag_ and BT_Ti–Ag–Ti_, respectively.

The lamellae were imaged in the STEM in tandem with energy dispersive X-ray spectroscopy (EDS), to obtain elemental mapping of the BT cross sections. Figure 4 shows dark-field images and EDS-generated compositional maps of the lamellae, demonstrating a clear difference between the two configurations. It is found that, unexpectedly, the BT_Ag–Ti_ structure is embedded in the SiO_2_ membrane, while the structure lacking an adhesion layer, BT_Ag_, is only negligibly embedded, if at all. (Additional EDS maps are shown in Figure S9, Figure S10, and Figure S11.)

**Figure 4:**
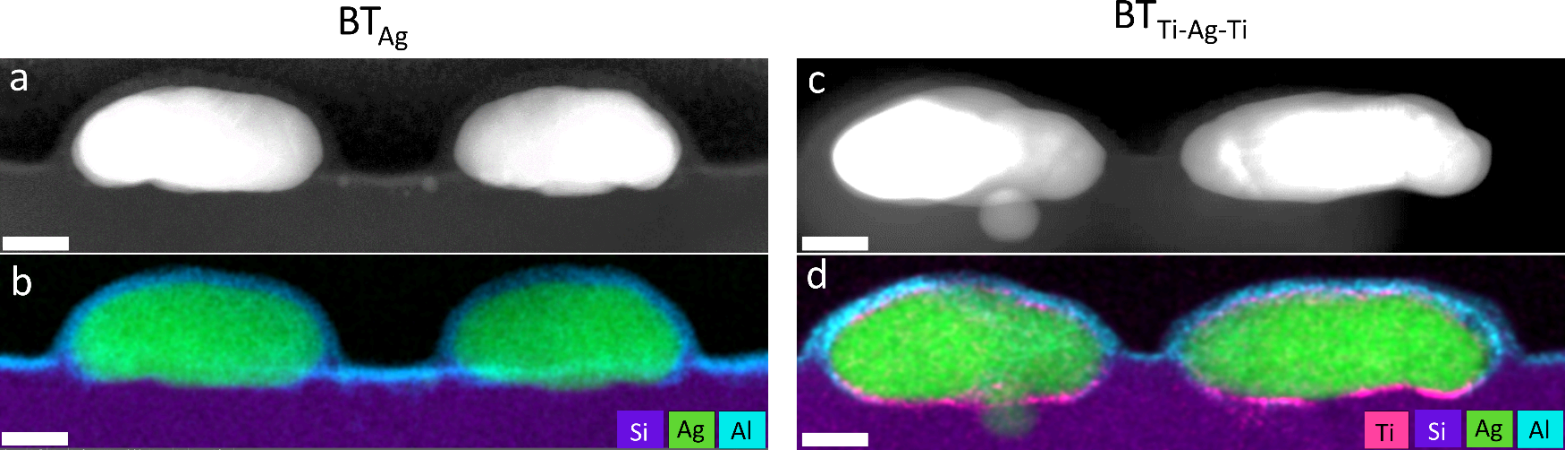
Dark-field TEM images of lamella cross sections of (a) BT_Ag_ and (c) BT_Ti–Ag–Ti_ and EDS compositional maps of Si, Ag, Al, and Ti elements for BT_Ag_ (b) and BT_Ti–Ag–Ti_ (d). Scale bar is 20 nm.

The lamellar analysis suggests that BTs deposited on a thin TiO_2_ layer get embedded in the silica substrate, so that a larger quantity of SiO_2_ is found in the gap region. The manifestation of narrower line widths and higher *Q* of longitudinal modes of the BTs might therefore be attributed to modification of the gap region by the addition of material with a higher refractive index.

### 2.5 Stronger Reduction of Radiative Efficiency of the Longitudinal Mode upon Embedding

To assess the role of the different decay channels in the line width narrowing, we compare the scattering intensity of the longitudinal mode between BT_Ag_ and BT_Ag–Ti_, measured under strictly identical experimental conditions. Here, the scattering intensity is defined as the area under the scattering curve, extracted from a Lorentzian fit to the scattering spectrum. Three such comparisons are presented in Figure S12a–c, where the normalized scattering intensity is plotted for both the BT_Ag_ and BT_Ag–Ti_ configurations. The mean and standard deviation of both the absolute and normalized scattering intensities for BT_Ag_ and BT_Ag–Ti_ across these three experiments are summarized in Table S2. We observe a decrease in the scattering intensity which indicates that the narrowing involves a reduction in the radiative decay rate, leading to a corresponding decrease in the radiative efficiency of the longitudinal mode. We note, however, that the relation between damping rate and scattering intensity is complex,^61^ so we treat this aspect here only qualitatively.

### 2.6 Discussion of Line Width Narrowing Mechanisms

These observations indicate that the longitudinal plasmon mode of BTs deposited on top of a thin (1 nm) Ti layer, which becomes oxidized during deposition, is significantly narrower than that of the same mode in comparable BTs directly deposited on SiO_2_. This behavior correlates with the partial embedding of the structures into the SiO_2_ substrate, which appears to be responsible for the observed line width narrowing. Importantly, line width narrowing is observed exclusively for the longitudinal mode, while no such effect is detected for the transverse mode or for nanoprisms. In parallel with the spectral narrowing, a comparison of scattering intensities points to a pronounced reduction in the radiative decay rate upon embedding for the longitudinal mode, whereas the transverse mode remains largely unaffected. This disparity indicates that the radiative decay is modified in a mode-selective manner, consistent with the observed narrowing of the resonance line width.

The observed mode-selective behavior is further illustrated by an analogous scenario in which the BTs are conformally coated with a higher-index material, demonstrating that modifications to the local dielectric environment—particularly within the gap—can similarly modulate the longitudinal-mode line width. In this case, Al_2_O_3_ deposited by ALD leads to decreased line widths compared to BT_Ag–Ti_ without Al_2_O_3_ (Figure 5), producing an effect of similar magnitude to the line width narrowing observed for BT_Ag–Ti_ relative to BT_Ag_. Because the Al_2_O_3_ layer conformally covers the entire BT surface, including the gap region, it effectively mimics embedding the structures in SiO_2_: the longitudinal mode experiences a more homogeneous, higher-index environment, resulting in narrower line widths. By contrast, when a Ti layer is deposited *in situ* immediately after the silver deposition and prior to the lift-off process, it coats only the top surfaces of the structures (Figure 3c). As a result, the coverage is incomplete and limited to the prism tops, while the BT gap remains unfilled with higher-index material. In this configuration, where only a small portion of the environment is modified, no line width narrowing is observed, whereas the effect is clearly recovered with a thin TiO_2_ adhesion layer. Consistent with the results shown in Figure 2, the transverse mode shows no line width narrowing or increase in quality factor upon deposition of Al_2_O_3_, as shown in Figure S13.

**Figure 5:**
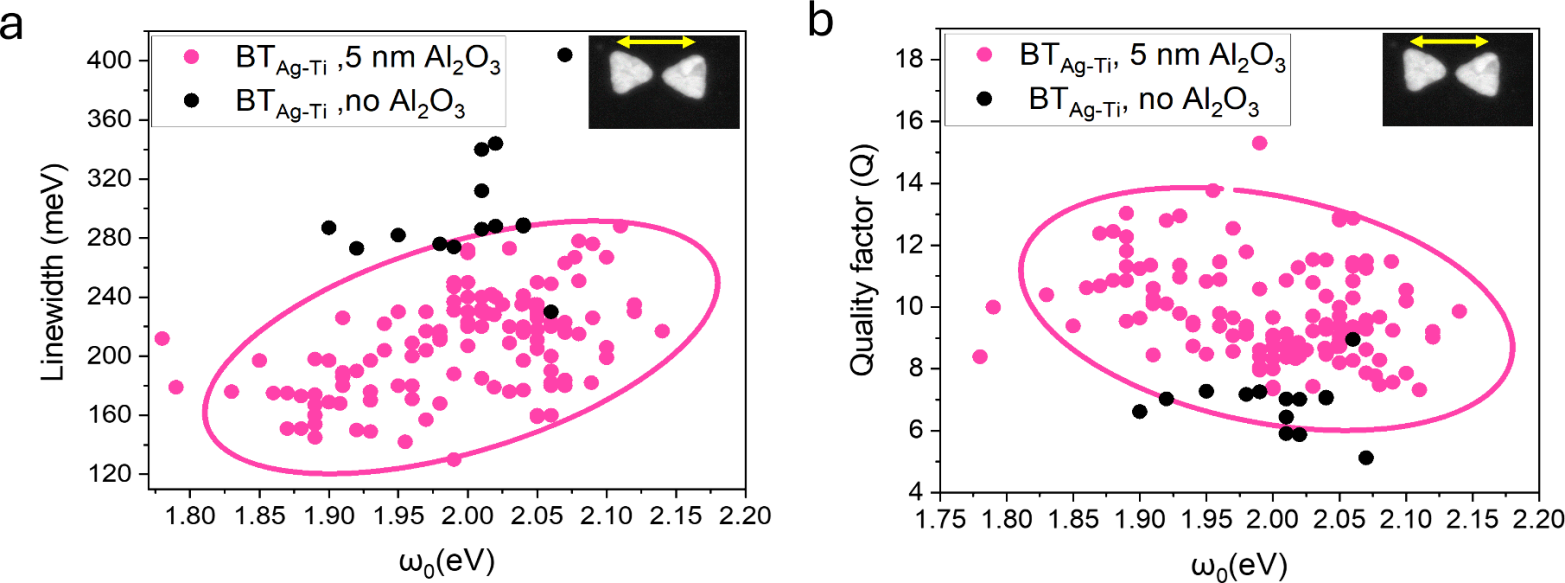
LSP spectral line widths (a) and quality factors (b) of the longitudinal mode as a function of LSP resonance frequency in energy units for BT_Ag–Ti_s without Al_2_O_3_ (black points) and with a 5 nm Al_2_O_3_ layer (pink points). Ellipse fits to BT_Ag–Ti_ s with Al_2_O_3_ layer are shown as pink curves. Yellow arrows indicate the direction of light polarization. Representative scattering spectrum of BT_Ag–Ti_ with no Al_2_O_3_ is shown in Figure S4f. Mean and standard deviation values of all data points are shown in Table S1 in the Supporting Information.

We next aim to shed light on the origin of the line width narrowing induced by embedding in SiO_2_. According to the model proposed by von Plessen et al., the radiative decay rate is expected to decrease quadratically with the plasmon resonance frequency.^40^ In our system, the addition of a TiO_2_ layer induces a moderate redshift of approximately 110 meV (Figure S14a). This enables direct comparisons at energy-matched points, where we observe a pronounced line width narrowing. Therefore, the frequency-dependent scaling of the radiative decay rate is effectively eliminated, and the redshift of the LSP resonance energy resulting from environmental modification does not play a role in the observed effect. The gap size is not the key factor behind this effect either, as BT_Ag_ and BT_Ag–Ti_ structures with similar gaps demonstrate different line widths (Figure S14b).

Embedding the BTs in a SiO_2_ membrane increases the effective refractive index experienced by the plasmonic modes, including within the gap region. Classically, increasing the refractive index of the surrounding medium is expected to broaden the radiative line width by enhancing the local density of optical states (LDOS), as described by Hartland et al.^49^ However, our experimental results reveal an opposite trend, indicating that in BT nanoantennas, the classical LDOS-induced broadening is outweighed by other mechanisms and plays only a secondary role. This behavior is further supported by numerical FDTD simulations, although in the simulations both modes exhibit line width narrowing. Simulated scattering spectra of two extreme configurations — BT_Ag_ on SiO_2_ without Al_2_O_3_ and BT_Ag–Ti_ embedded in SiO_2_ and coated with Al_2_O_3_ — are shown in Figure S15. Although experimentally this effect is observed only for the longitudinal mode, the trend of spectral narrowing accompanied by a decrease in scattering intensity is consistent with the simulations (see details in the Supporting Information).

This radiative line width narrowing in the BT_Ag–Ti_ system aligns with the theoretical framework of Grigorchuk, which attributes suppressed radiative damping to the polarization of the high-index dielectric layer.^62^ In this picture, the surrounding medium polarizes in opposition to the electron oscillation within the metal, effectively “screening” the transition dipole moment. As a result, the radiative decay rate decreases with increasing refractive index of the surrounding medium—an effect that Grigorchuk showed to be significantly amplified in nonspherical geometries. Although Grigorchuk’s original models focus specifically on spheroidal particles, characterized by two equal semiaxes and a third unequal axis, the underlying physical principles can be qualitatively extended to the extreme field confinement present in the BT nanogap. The classical LDOS-induced broadening predicted by Hartland et al.^49^ and the dielectric screening mechanism proposed by Grigorchuk^62^ therefore represent two competing effects. Our experimental observation of radiative line width narrowing suggests that, for the BT geometry studied here, the classical LDOS-induced broadening might be outweighed by dielectric screening.

Experimentally, the fact that radiative narrowing is observed exclusively in the longitudinal mode—and is absent in both the transverse mode and individual nanoprisms—is perhaps the most striking finding of this study. This mode selectivity is particularly surprising given that the simulations do not capture this behavior. Instead, in the FDTD simulations both modes exhibit line width narrowing, with the transverse mode showing a larger reduction (38%) than the longitudinal mode (23%), along with a more pronounced decrease in scattering intensity (Figure S15). In contrast, experimentally the transverse mode shows neither narrowing nor a reduction in scattering intensity. This discrepancy highlights that the simulations, which assume idealized particle geometries and perfectly coherent interactions with the dielectric layers, do not fully capture all factors present in the real system. Understanding the precise physical origin of this mode selectivity will require further study, potentially including more realistic modeling of structural imperfections and dielectric inhomogeneity.

## 3 Conclusions

We have demonstrated that partial embedding of BT nanoantennas in SiO_2_, enabled by a thin Ti adhesion layer, leads to a pronounced and mode-selective narrowing of the longitudinal plasmon line width, accompanied by reduced scattering intensity and suppressed radiative decay.

Our results show that nanoscale modification of the local dielectric environment—particularly within the nanogap—provides an effective route for engineering radiative damping in plasmonic systems. The observed line width reduction cannot be explained by conventional LDOS-based arguments and instead points toward a dielectric screening mechanism that competes with radiative enhancement effects.

This spectral line width narrowing has direct implications for plasmonic applications that rely on sharp resonance and strong near-field confinement. The narrower line widths of embedded or gap-filled BTs enhance spectral resolution and improve the figure of merit in refractive-index sensing.^63^ In addition, reduced radiative damping increases the fraction of energy dissipated through nonradiative channels which can be advantageous for photothermal conversion.^64^ Importantly, line width reduction is highly beneficial for light–matter interactions in the strong-coupling regime, where the visibility of Rabi splitting depends on the plasmon line width relative to the emitter–plasmon coupling strength.^10,48,65–67^ Previous work has already shown that high-Q silver BTs can reach single-particle strong coupling, and the longitudinal-mode narrowing observed here should further enhance the visibility of coupling in spectra.^48^ These results therefore strengthen the potential of BT plasmonic cavities for room-temperature cavity-QED and hybrid plasmon–emitter devices in which dissipative properties can be selectively engineered.

## 4 Experimental Section

### 4.1 Materials

SiO_2_ membrane (pore size ∼ 100 nm) was purchased from TedPella, Inc. Poly­(methyl methacrylate) (PMMA, Mw ≈ 950 k, 2% in anisole) was used as the electron beam lithography resist. Methyl isobutyl ketone (MIBK) and isopropyl alcohol (IPA) were used as a 1:3 developer solution for PMMA patterning. Remover PG and acetone were used as the resist remover for lift-off procedure. All those chemicals were purchased from Kayaku, Inc.

Titanium (99.995%) and silver (99.999%) were purchased from Angstrom Engineering Ltd. and used as received. Trimethylaluminum (TMA, 99.999%) was used as the aluminum precursor for ALD and was obtained from STREM Chemicals, Inc.

### 4.2 Procedures

#### 4.2.1 Sample Fabrication

The substrates used in this study were SiO_2_ membranes, 18 nm thick. We spin-coat PMMA resist at 4000 rpm to form a 60 nm thick layer followed by baking on a hot plate at 180 C^0^. BT structures are then exposed in the resist by electron beam lithography (e_Line Plus RAITH), using an accelerating voltage of 30 keV and an aperture size of 10 μm, yielding a beam current of 40 pA. The overall dose ranged between 600 to 1200 μC/cm^2^, resulting in structures with gap sizes ranging between a few nm to 40 nm. We developed the sample in a MIBK/IPA (1:3) resist developer for 30 s, followed by an Isopropanol stopper for 30 s.

Ti and Ag depositions were performed in an evaporator system (ODEM ltd.) with a base vacuum of 5·10^–8^ mTorr. The deposition rates of Ti and Ag are fixed to 0.01 nm/sec and 0.05 nm/sec, respectively. For lift-off, the sample is dipped in a solution of remover PG for 10 min followed by acetone rinsing to wash out the PMMA resist. To remove the risk of breaking the membrane during lift-off due to mechanical stress, we avoid sonication. Also, the remover PG solution is kept at room temperature during the lift-off procedure to avoid degradation of the BTs. For protecting the Ag layer from oxidation and degradation, we deposit a 5 nm thick Al_2_O_3_ layer on top, using an ALD system (Fiji Plasma). EDS mapping revealed no evidence of bulk silver oxidation following this procedure.

#### 4.2.2 TEM Lamella Preparation

We prepare cross-sectional lamellas of the BT structures for further TEM analysis using a modified lift-out procedure within a FIB microscope (Helios-600 Thermo Fisher Scientific, Massachusetts, USA).

The chip was first coated with a layer of amorphous carbon of about 5 nm from both sides in a Safematic CCU-010 Coating device (Safematic GmbH, Switzerland) to improve conductivity of the sample. Then, a thicker patch of carbon layer (∼100 nm) was deposited locally over the SiO_2_ membrane, using e-beam induced chemical vapor deposition (CVD) by a gas injection system within the FIB chamber. An additional patch of carbon was deposited on the region of interest at the back side of the chip.

In order to improve the accuracy of the lamellar positioning during the final polishing, we deposit guidelines above the protective layer of carbon, particularly “x”-shaped platinum lines, 50–60 nm thick, with a crosshair in the region of the center of the BT structures. Additional patches of protective layers of carbon (∼0.5 um) and Pt (∼1 um) are deposited above the Pt guidelines. To complete protection of the region of interest, additional layers of carbon (0.5–0.6 um) and Pt (0.9–1 um) are deposited above the guide markers, using ion-beam induced CVD.

The prepared region is cut out from the chip, extracted using a micromanipulator, mounted on a copper lift-out semigrid, and then finally polished down to 50–70 nm.

### 4.3 Characterization Methods

#### 4.3.1 Dark-Field Spectroscopy

Scattering spectra of single BTs are measured with an inverted microscope equipped with a dark-field condenser, a 75 W xenon lamp (Olympus), and a 100× oil immersion objective with NA = 1.30. A SpectraPro-150 spectrograph with a 1200 g/mm grating (Acton) and a Newton spectroscopy CCD camera (Andor Technology) are used to disperse scattered photons and register spectra. Raw spectra are smoothed using a Fourier low-pass filter. Prior to the spectral measurements, the camera is calibrated in reference to the spectrum of fluorescent dyes. Polarization of the excitation light is controlled by a combination of a polarizer and a selecting sector, such that essentially only s-polarized light reached the sample. Schematic of the dark-field microspectrometer is shown in Figure S16.

Scattering spectra is acquired from individual BT antennas under dark-field illumination. For each antenna, a region of interest (ROI) is selected on the camera image to include the BT structure, and the corresponding spectrum is recorded. To account for background contributions, two additional ROIs are selected in adjacent areas of the substrate, away from the nanostructure, and their spectra are acquired. The background spectra are averaged and subtracted from the antenna spectrum. The resulting spectrum is then normalized by dividing by the lamp spectrum, yielding the corrected scattering response of the BT.

STEM images and analytical EDS maps are acquired in a double aberration-corrected electron microscope (Themis-Z, Thermo Fisher Scientific Electron Microscopy Solutions, Hillsboro, USA) at an accelerating voltage of 200 kV. STEM images are recorded with a Fischione Model 3000 detector and a bright field (BF) detector (Thermo Fisher). The images are obtained with an electron probe with a convergence angle of 21 mrad and a primary beam current of less than 50 pA. EDS hyperspectral data are obtained at a beam current of 200 pA. with a Super-X silicon drift detector (SDD) and quantified with the software Velox (TFS) through background subtraction and spectral deconvolution

#### 4.3.2 XPS

XPS measurements are performed in a Kratos AXIS-Ultra DLD spectrometer, using its monochromatic Al kα source at a relatively low power, 15–75 W. We preform XPS on BT structures with three layer-by-layer configurations, as shown in Figure S1a–c, as well as on a similar selection of flat samples. Specifically for the study of Ti oxidation states, samples free of the top Al-oxide and with thinner Ag BTs, 8 and 10 nm instead of 25 nm, are used, such as to better probe the thin Ti layer underneath by this surface-sensitive technique. In order to gain signal, these samples are fabricated as matrices of BTs, separated by only 1 μm, and covering an overall area of 300 μm^2^.

Although charging artifacts are expected to be challenging due to the highly insulating nature of the substrate, the presence of the metallic Ag islands surrounded by domains of finite surface conductivity allows us to obtain spectra with only small charging shifts by (1) employing a top contact just slightly away from the matrix of BTs; and (2) using the X-ray source with low power. To complement these measurements, a higher X-ray power was also employed, and an electron flood gun (eFG) was activated, such as to gain improved statistics under well-controlled charging conditions. These complementary measurements further allow the extraction of useful structural information, based on the analysis of line-shift magnitudes.^68,69^

#### 4.3.3 XRD

In-plane XRD measurements are performed on a SmartLab (Rigaku, Japan) diffractometer equipped with a rotating Cu anode operating at 45 kV and 200 mA and with a HyPix-3000 two-dimensional detector operating in 0D mode with an incident slit of 0.1 mm. During the measurement, theta and 2theta angles are 0.3 deg. A quasi-parallel X-ray beam is formed by a multilayer mirror (CBO attachment, Rigaku) and shaped by 0.5^0^ in-plane soller slits before and after the sample. The detector was operated with the scattering and receiving slits open. X-ray diffraction (XRD) patterns are collected with an offset of 3 degrees on TTRAX III diffractometer (Rigaku, Japan) equipped with a rotating anode at a voltage of 50 kV and a current of 200 mA. A monochromator is installed before the scintillator detector. Sollers of 5 deg are installed before and after the sample. Analysis is done using the software Jade (MDI).

XRD is applied on two Ag films: (1) 25 nm Ag films deposited directly on SiO_2_ substrate (2) 25 nm silver film deposited on 1 nm Ti adhesion layer, using SiO_2_ substrate. We maintain the same deposition parameters as used for the experiment and XPS analysis.

## Supplementary Material



## Notes

XPS spectra; quality factor values and line widths of BT_Ag_ and BT_Ag–Ti_ across different substrates; representative scattering spectra under various geometries and interface conditions; spectral line widths and resonance energies of BTs and nanoprisms across the full frequency range; scattering spectra with thicker Ti adhesion layers; XRD spectra; STEM bright-field cross-sectional images; EDS compositional maps; scattering intensities; spectral line widths and quality factors of the transverse mode with and without Al_2_O_3_; gap dependence of resonance energy and line width; numerical FDTD simulations of longitudinal and transverse modes; schematic of the dark-field microspectrometer setup; tables of line widths, quality factors, resonance frequencies, and scattering intensities (PDF)
