# Supplementary material for: Selective Narrowing of the Bonding Modes of Plasmonic Nanoantennas
Source: ACS Appl Mater Interfaces. 2026 Jun 2;18(23):32849–58. doi: 10.1021/acsami.6c05142 (PMC13288403; doi:10.1021/acsami.6c05142)
Supplement: Supplementary file 1 [file am6c05142_si_001.pdf]

Supporting Information

Selective narrowing of the bonding modes of plasmonic nanoantennas

Ora Bitton[1,*], Lothar Houben[1], Hagai Cohen[1], Katya Rechav[1], Sigal Keshet[1], Anna Kossoy[1], Marek Patočka[1], Eran Mishuk[1], Bar Cohn[2,3,4], Lev Chuntonov[2,3,4], Alexander Vaskevich[5] and Gilad Haran[5]

[1] Department of Chemical Research Support, Faculty of Chemistry, Weizmann Institute of Science, Rehovot 7610001, Israel

[2] Schulich Faculty of Chemistry, Technion–Israel Institute of Technology, Haifa 3200003, Israel

[3] Solid State Institute, Technion–Israel Institute of Technology, Haifa 3200003, Israel

[4] The Helen Diller Quantum Center, Technion–Israel Institute of Technology, Haifa 3200003, Israel

[5] Department of Chemical and Biological Physics, Faculty of Chemistry, Weizmann Institute of Science, Rehovot 7610001, Israel

*Corresponding author: ora.bitton@weizmann.ac.il

Fig. S1

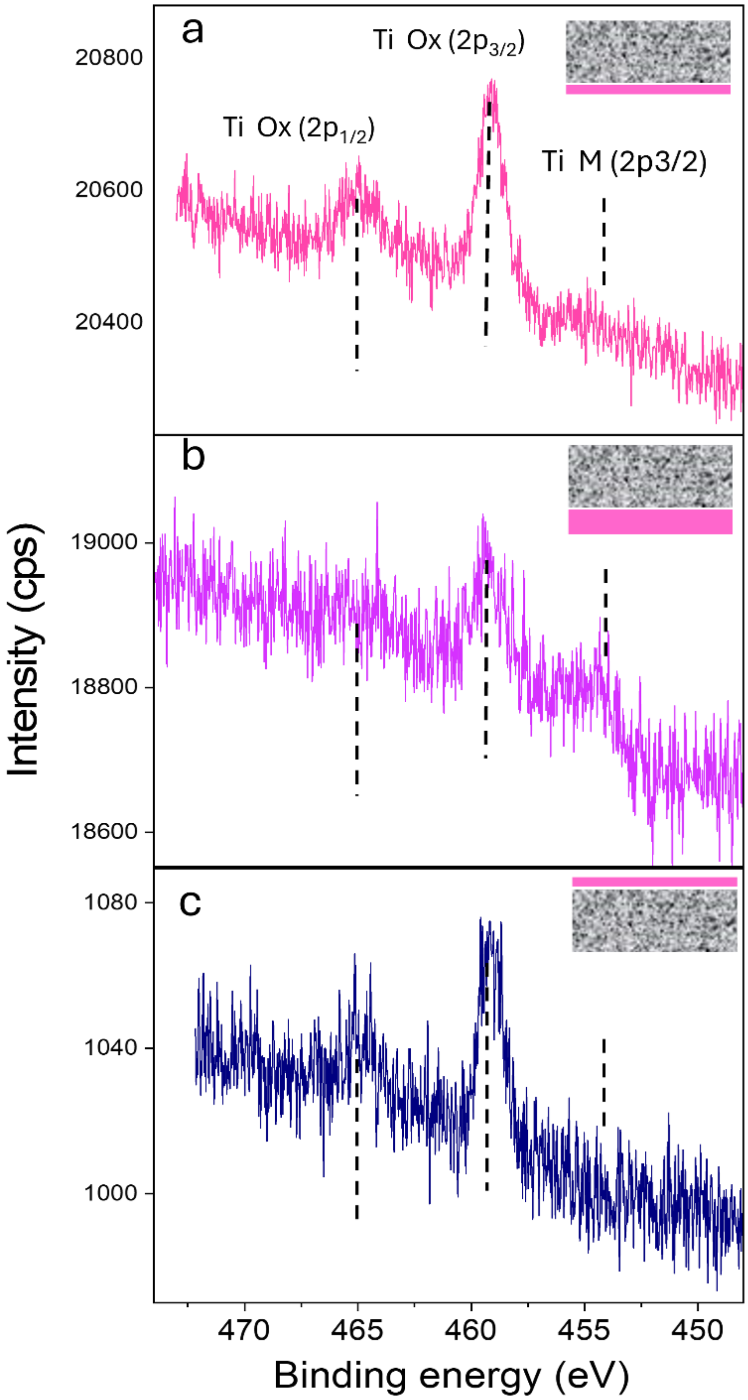

a
b
c
Ti Ox (2p3/2)
Ti Ox (2p1/2)
Ti M (2p3/2)
20800
20600
20400
19000
18800
18600
1080
1040
1000
Intensity (cps)
470
465
460
455
450
Binding energy (eV)

Figure S1: XPS Ti 2p spectra of samples with different layer-by-layer configurations: (a) Ti layer of a 1 nm nominal thickness underneath the Ag BTs; (b) same as panel a, however with a Ti layer of 5 nm nominal thickness; (c) 1 nm Ti layer deposited just on top of the Ag-BTs. The insets show schematically the location of the Ti layer (pink) with respect to the silver (grey). Two literature binding energies of the $2p_{3/2}$ peak are indicated, 459.1 eV and 453.9 eV for TiO_2 and metallic Ti, respectively. Note the apparent Ti oxidation in all samples. Metallic Ti signal (broadened due to intermediate oxidation states) is partially preserved in the thicker layer, panel b. However, it nearly vanishes in the other two samples.

Fig. S2

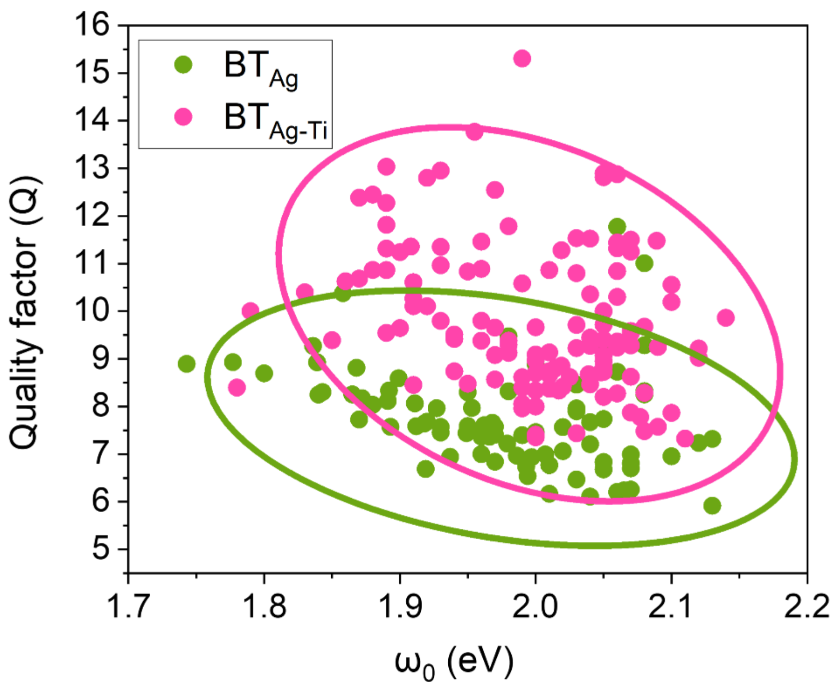

Figure S2: Quality factor values of BT$_{Ag}$s and BT$_{Ag-Ti}$s as a function of plasmon resonance frequency in energy units.

Fig. S3

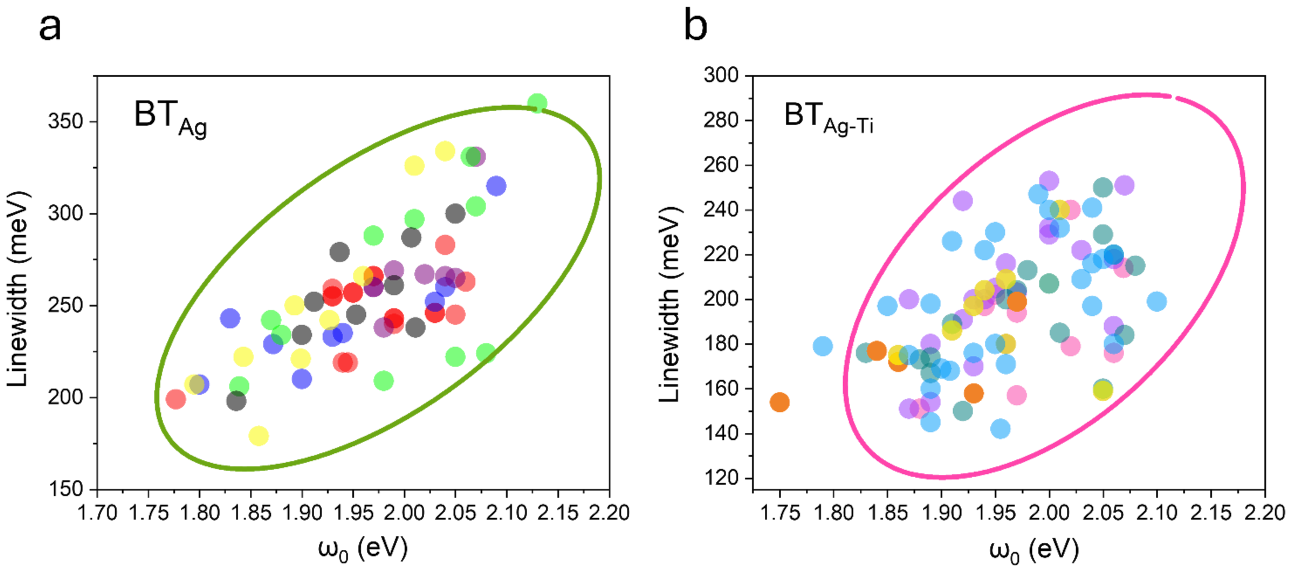

Figure S3. Linewidths of BT_{Ag}s and $BT_{Ag\text{-}Ti}$s are measured on different substrates, with each substrate represented by a distinct color. The green and pink curves show the elliptical fits taken from Fig. 1c. The linewidths obtained from the individual substrates span the full area of the ellipse, demonstrating that the observed narrowing effect is not substrate-dependent.

Fig. S4

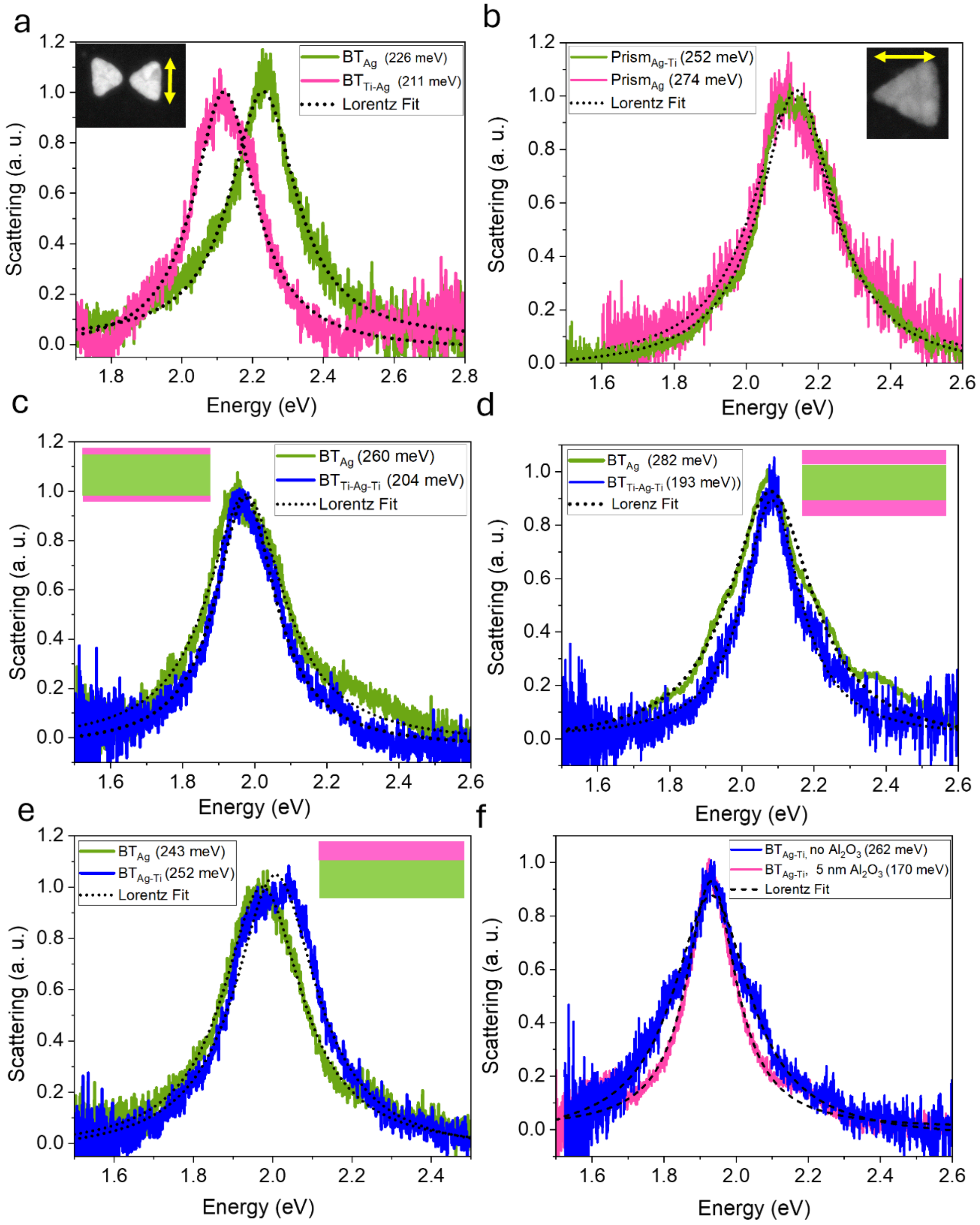

Figure S4: Representative scattering spectra of (a) transverse mode of BT_{Ag} (green) and BT_{Ag-Ti} (pink). (b) $prism_{Ag}$ (green) and $prism_{Ag-Ti}$ (pink). (c) Longitudinal mode of $BT_{Ti-Ag-Ti}$ with nominal 0.2

nm deposited Ti (blue) and BT$_{Ag}$ (green). (d) Longitudinal mode of BT$_{Ti\text{-}Ag\text{-}Ti}$ with nominal 1 nm deposited Ti (blue) and BT$_{Ag}$ (green). (e) Longitudinal mode of BT$_{Ti\text{-}Ag}$ with nominal 1 nm deposited Ti (blue) and BT$_{Ag}$ (green). (f) Longitudinal mode of BT$_{Ag\text{-}Ti}$ without Al_2O_3 layer (blue) and BT$_{Ag\text{-}Ti}$, with Al_2O_3 (pink). Dotted black curves are Lorentzian fits from which FWHM values were extracted and are specified in the legends. Yellow arrows indicate the direction of light polarization. The schemes in panels c,d,e correspond to the BT configuration associated with the blue curve.

Fig. S5

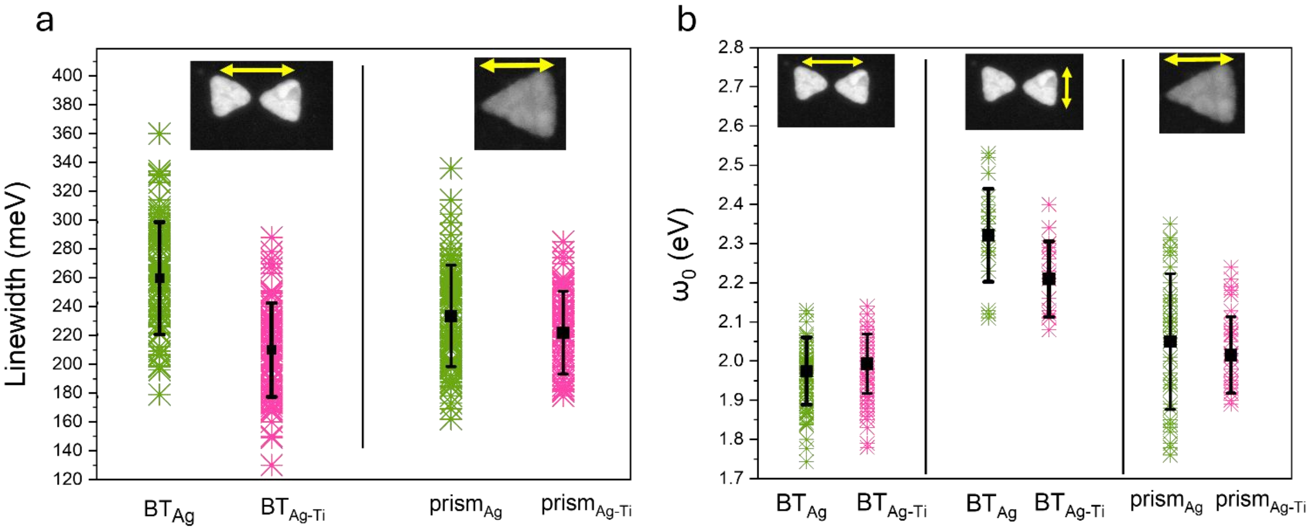

Figure S5: (a) Spectral linewidths of longitudinal modes of all BT$_{Ag}$s and BT$_{Ag\text{-}Ti}$s (left) and all prism$_{Ag}$s and prism$_{Ag\text{-}Ti}$s. (b) LSP resonance energies ω_0 of longitudinal modes (left) and transverse modes (center) of all BT$_{Ag}$s and BT$_{Ag\text{-}Ti}$s and of all prism$_{Ag}$s and prism$_{Ag\text{-}Ti}$s (right). Yellow arrows indicate the direction of light polarization. Black squares and black error bars are the mean values and the standard deviations respectively. See values of mean values and standard deviations in Table S1.

Fig. S6

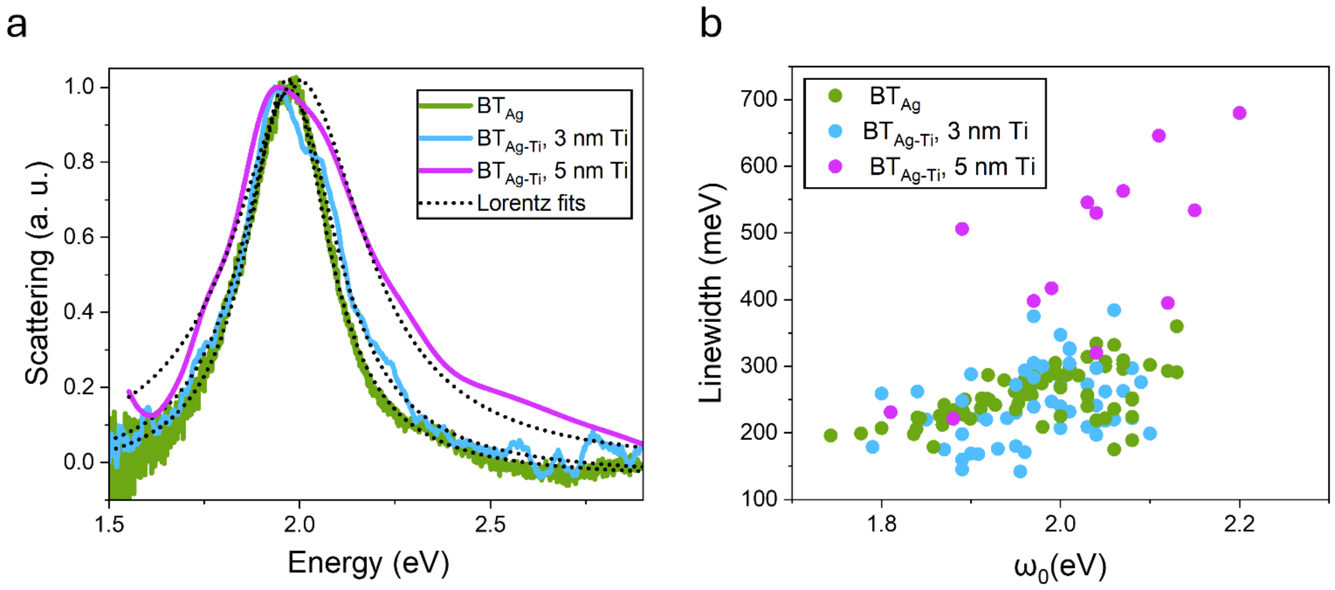

Figure S6: (a) Scattering spectra of BT_{Ag} and $BT_{Ag\text{-}Ti}$ with 3 and 5 nm deposited Ti adhesion layers demonstrating LSP spectral linewidths of 257, 305 and 417 meV, respectively. (b) Spectral linewidths for multiple BTs.

Fig. S7

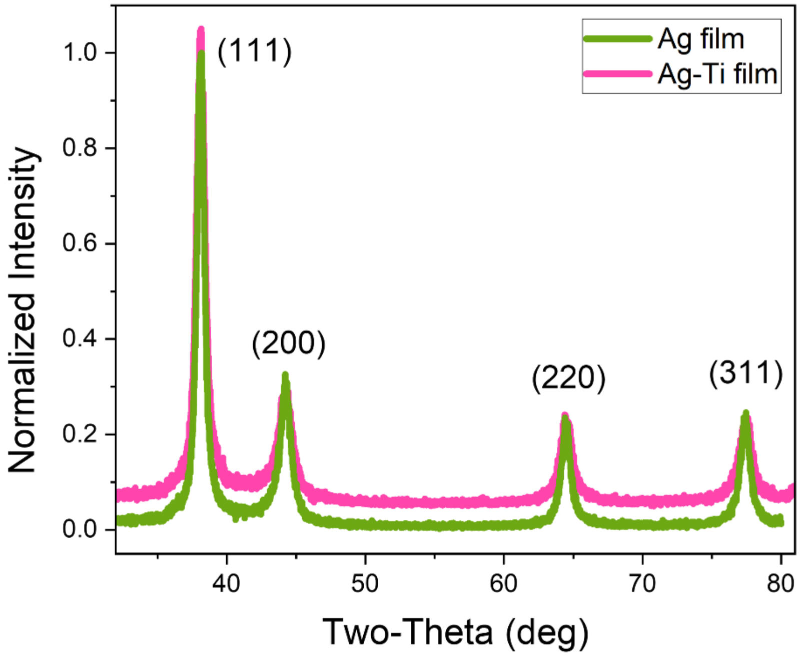

Figure S7: In-plane diffraction pattern of the film with and without Ti layer at the interface. The crystals are randomly oriented with four typical directions, indicated in the figure.

Fig. S8

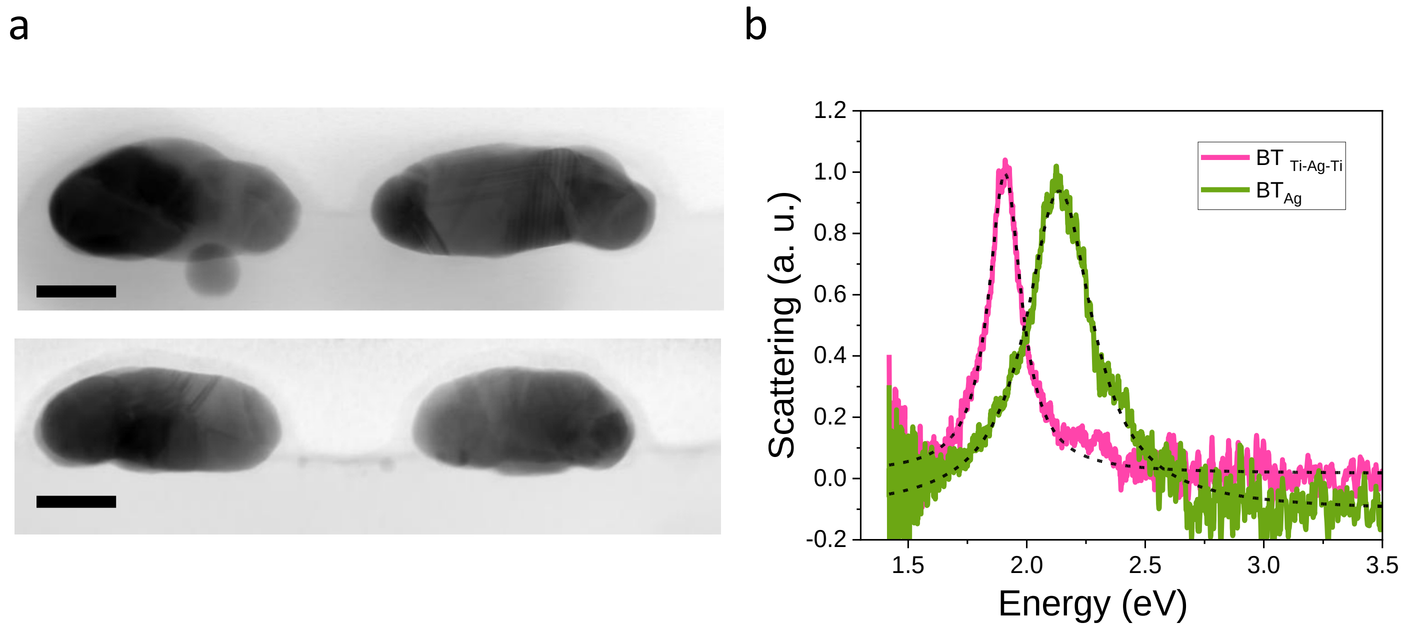

Figure S8: (a) High-resolution STEM bright field images of the cross-sectional two BTs, $BT_{Ti\text{-}Ag\text{-}Ti}$ with 0.2 nm nominal Ti thickness (upper image) and BT_{Ag} (bottom image). (b) The scattering spectra of these BTs demonstrating resonance frequency ω_0 of 2.14 eV and 1.9 eV and a spectral linewidth of 343 meV and 168 meV for BT_{Ag} and $BT_{Ti\text{-}Ag\text{-}Ti}$, respectively.

Fig. S9

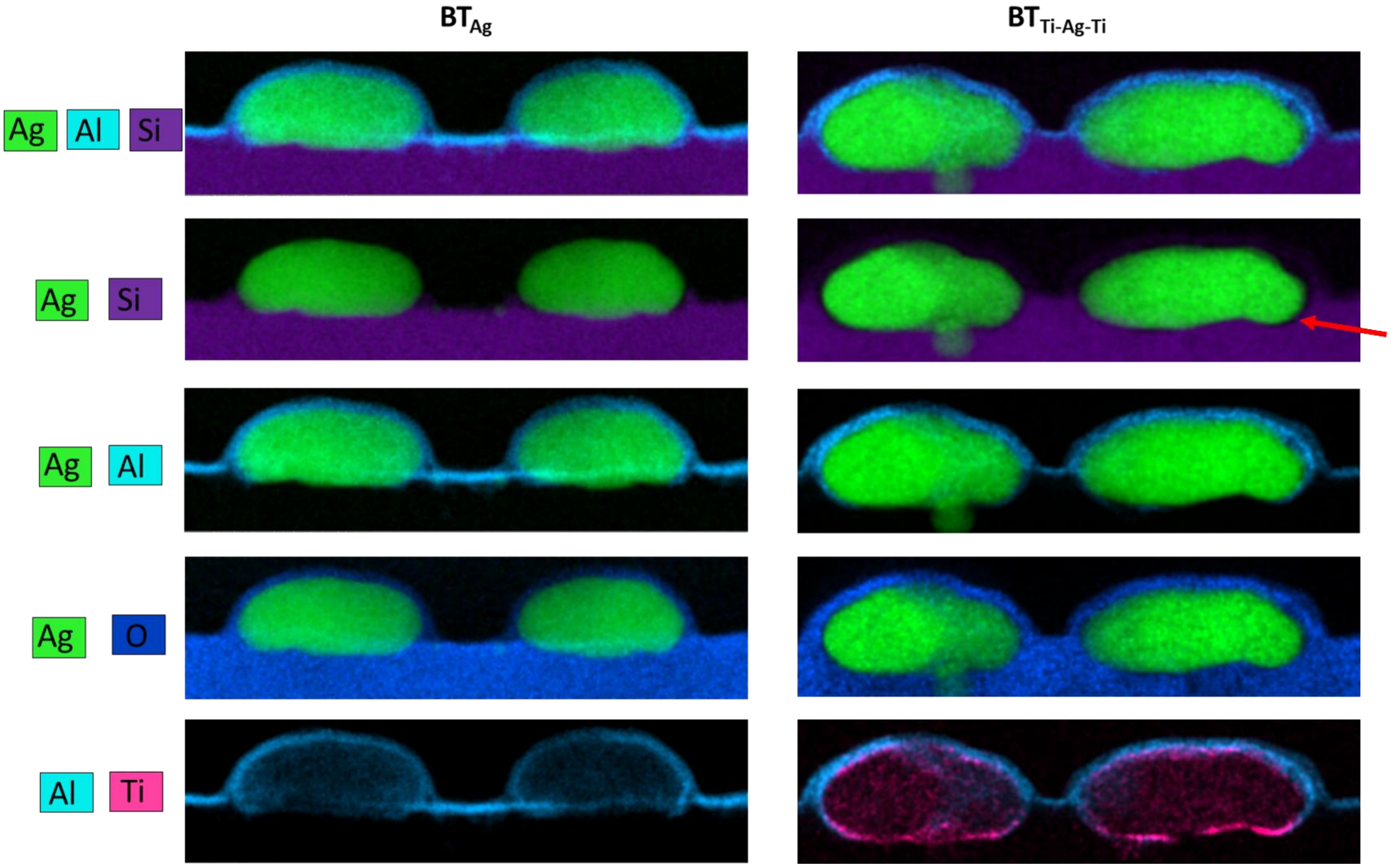

Figure S9: EDS-generated compositional maps indicating the distribution of several sets of elements in BT_{Ag} and $BT_{Ti\text{-}Ag\text{-}Ti}$ configurations. The overlapped Ag and Si EDS maps of the $BT_{Ag\text{-}Ti}$ demonstrate a noticeable gap at the interface between the Si and the Ag (indicated by red arrow), while EDS maps of Ag-Ti and Ag-O indicate that the silver has a sharp interface both with the Ti and the oxygen. The observation that the oxygen map overlaps with the Ti map in the gap region at the interface is in accordance with the fact that the Ti at the interface is oxidized, as was mentioned in the main text and validated by XPS. Spurious Ti counts in the interior of the Ag prisms in the EDS elemental map on the lower right are an artefact related to the background noise that occurs when quantifying the naturally low number of counts in a spectrum corresponding to a single sampling point in the map. See more details in Fig. S10 and Fig. S11.

Fig. S10

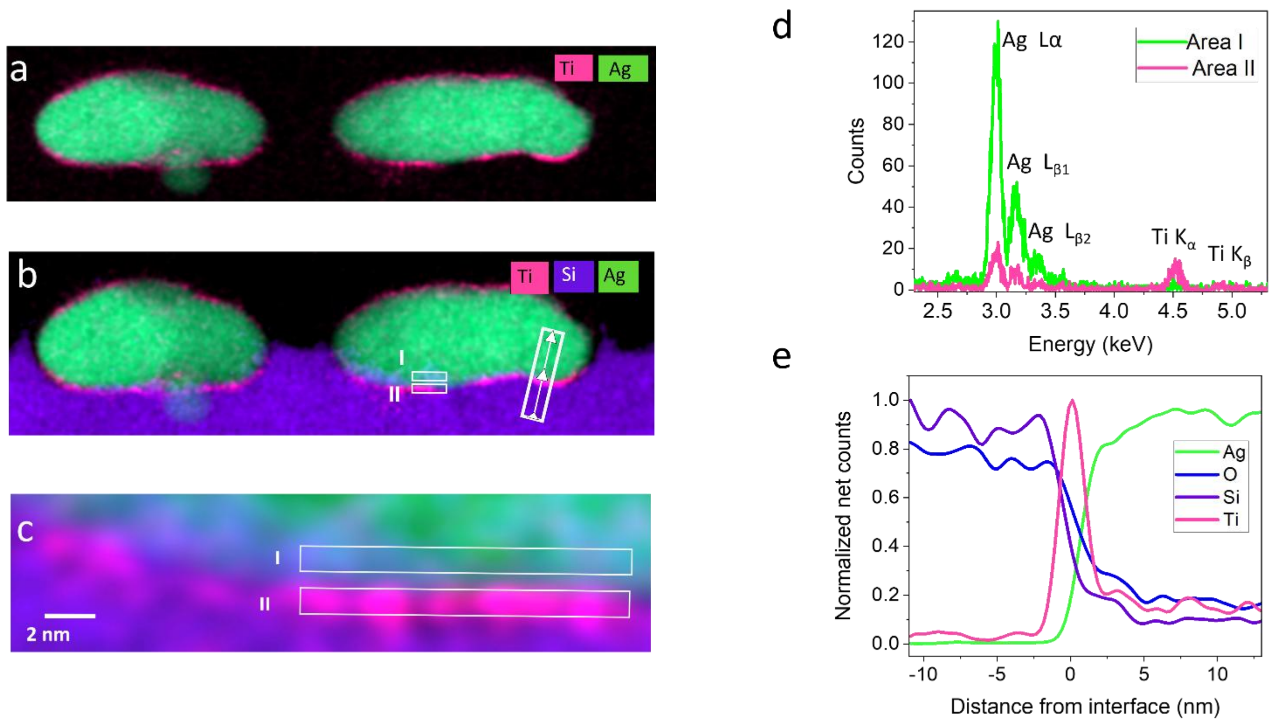

Figure 10: (a) EDS map of Ti-Ag. (b) EDS map of Ti-Si-Ag. Quantitative spectral data were extracted from small areas adjacent to the interface (areas I and II marked in panels b and c). The local spectra on the interface layer and the region just above and inside the Ag prism show a strong decrease of the Ti signal inside the Ag grain (panel d). (e) A line profile of elemental counts across the interface, indicated by a white arrow in b. The Ti intensity along the profile manifests a clear peak corresponding to the Ti layer (pink curve) with a linewidth of 2 nm (FWHM). While Ti intensity decreases, Ag intensity starts to increase, which implies that Ti is separated from the Ag film. It is clearly observed that the apparent Ti concentration is drastically reduced inside the Ag prisms, and no alloy phase is formed. Already the first atomic Ag layer hardly contains Ti, in agreement with a low solubility of Ti in Ag in the range of a few at %. The O profile (blue curve) is shifted with respect to the Si profile but follows the Ti curve. This strengthens the observation obtained from the EDS maps (Fig. S9) that O appears together with Ti, implying the presence of oxidized Ti rather than elemental titanium. The cross-sectional analysis, besides supporting the full oxidation of the Ti, reveals that the TiO_2 layer is separated from the Ag film and no alloys form.

Fig. S11

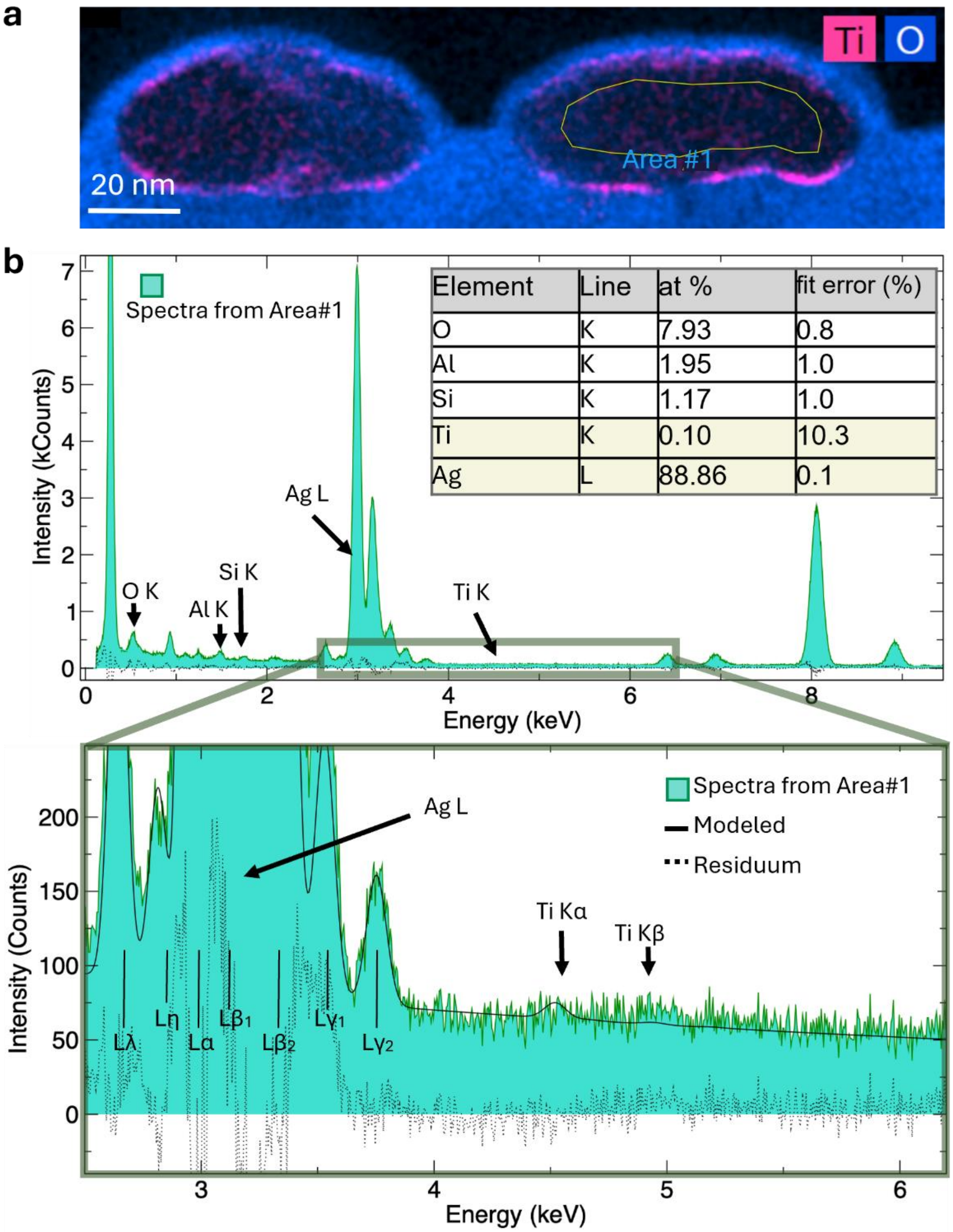

Element	Line	at %	fit error (%)
O	K	7.93	0.8
Al	K	1.95	1.0
Si	K	1.17	1.0
Ti	K	0.10	10.3
Ag	L	88.86	0.1

Figure S11: Phase purity of the Ag prisms. (a) EDS elemental map of Ti, and O, reproducing the data shown in Fig. S9. (b) Sum-spectrum over the interior of an Ag prism, marked as 'Area 1' in (a), and the related elemental quantification. The Ti concentration in the interior of the prism drops below the sensitivity limit of the EDS detection < 0.2 at %. A low concentration of Si, O, and Al, indicated in the quantification table, originates from the tip area of the prism, where the cross-sectional FIB lamella is geometrically thicker than the bowtie, thus the summed spectra partially

include signal from the surrounding media. The lower part of panel (b) shows a magnified view of the cumulative spectrum, 30x magnified on the intensity scale. There is no significant signal intensity above background noise at the Ti K region, consistent with the low concentration at the sensitivity limit. The elemental quantification on the EDS spectra cumulated across the volume of the Ag prism further shows the absence of Ti within the sensitivity of detection. The Ti level in the prisms is lower than 0.2 at %. We thus have no indication for Ti diffusion into Ag or the formation of an AgTi binary phase.

Fig. S12

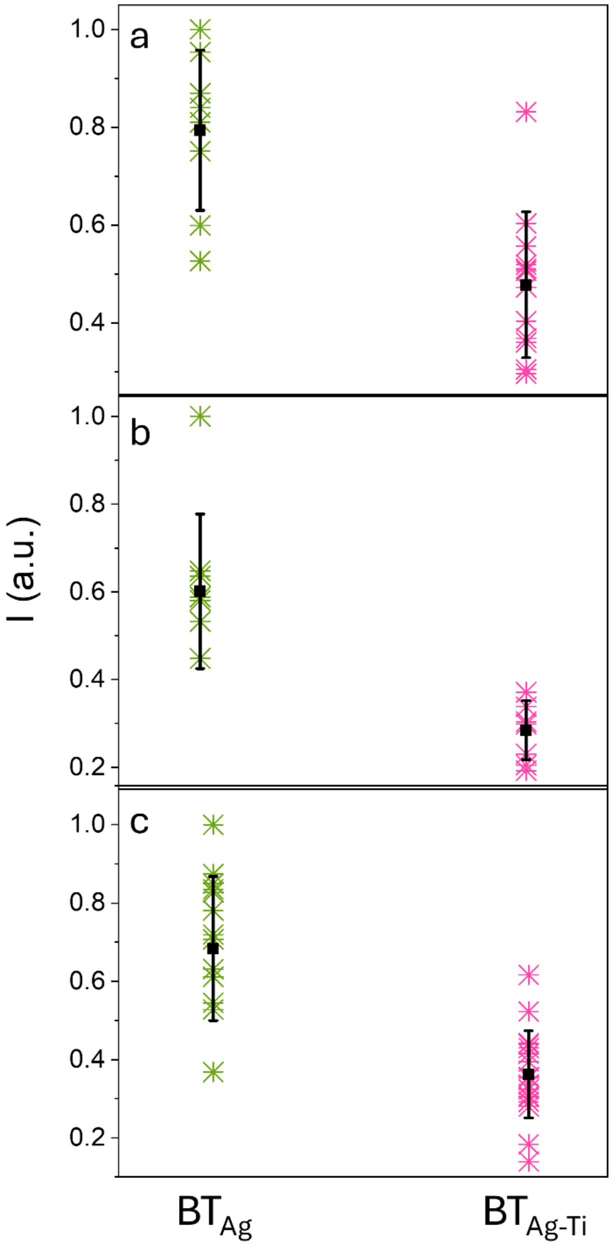

Figure S12: Comparison of the scattering intensities of BT$_{Ag}$ and BT$_{Ag–Ti}$. (a-c) Scattering intensity values for the two configurations BT$_{Ag}$ and BT$_{Ag–Ti}$ in groups of bowties measured under identical conditions. The scattering intensity is defined as the area under the scattering curve, extracted from a Lorentzian fit to the measured spectrum. Black squares and black error bars are the mean values and the standard deviations respectively. The mean and standard deviation of the data points (absolute and normalized values) are provided in Table 2. For each group, the intensity

distributions were statistically compared using a two-sample Kolmogorov–Smirnov test, yielding statistically significant differences in all three cases ($p = 3.78 \times 10^{-3}$, 1.707×10^{-4}, 8.6186×10^{-6}), indicating that the distributions are unlikely to originate from the same underlying population.

Fig. S13

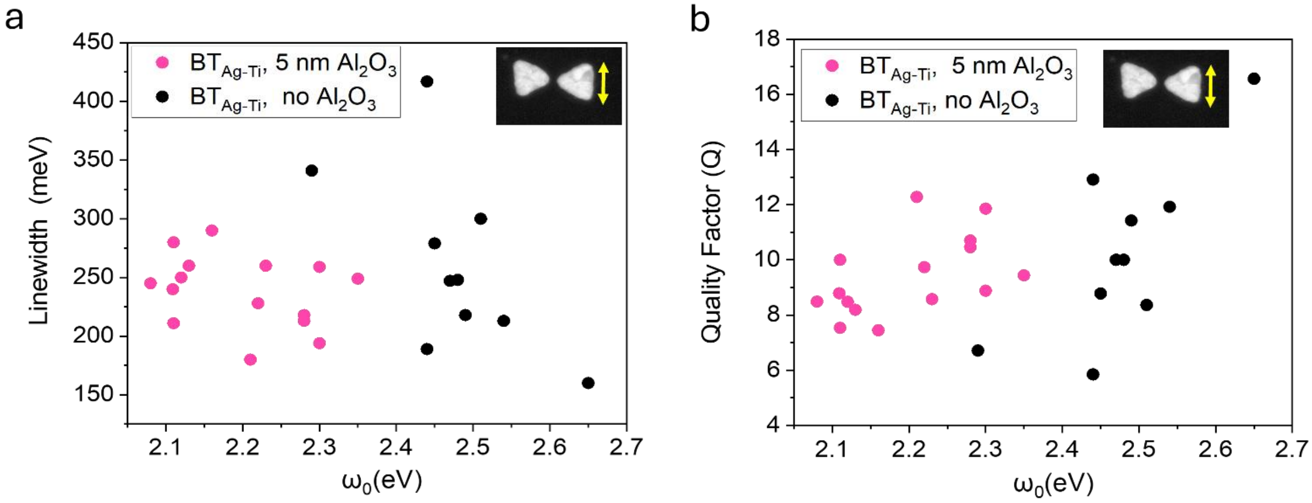

Figure S13. LSP spectral linewidths (a) and quality factors (b) of the transverse mode as a function of LSP resonance frequency in energy units for $BT_{Ag–Ti}$s without Al_2O_3 (black points) and with a 5 nm Al_2O_3 layer (pink points). Yellow arrows indicate the direction of light polarization. Mean and standard deviation values of all data points are shown in Table S1 in the supplementary information.

Fig. S14

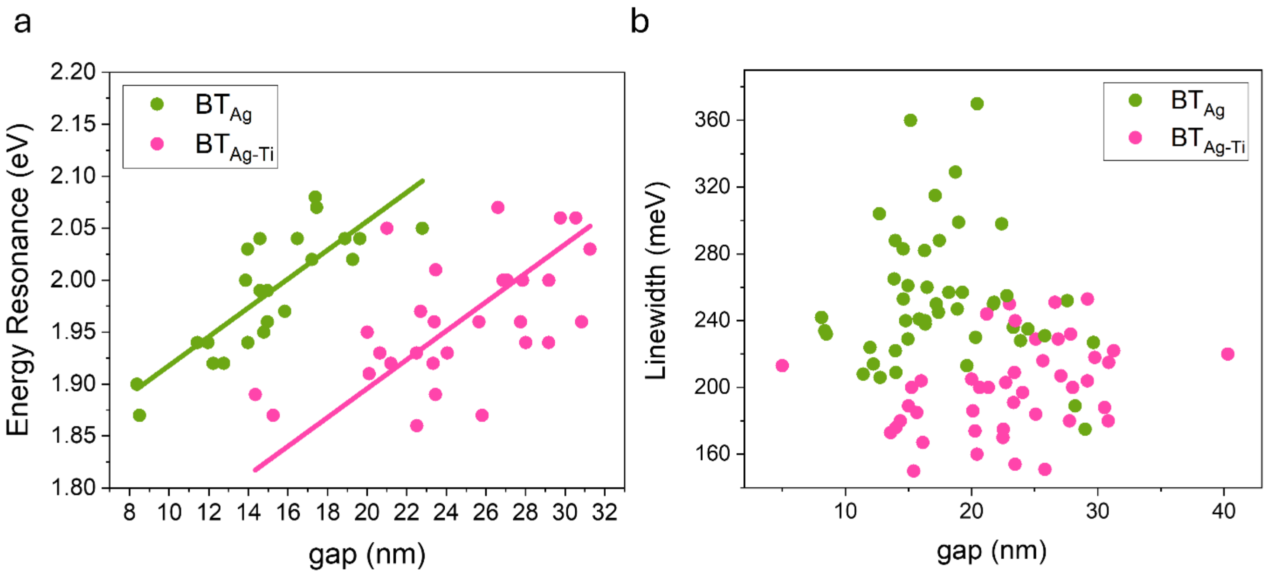

Figure S14: Gap dependence. (a) Energy resonance of BT_{Ag}s (green spots) and $BT_{Ag\text{-}Ti}$s (pink spots) as a function of gap size. Lines are linear fits. (b) LSP spectral linewidth values of BT_{Ag}s (green spots) and $BT_{Ag\text{-}Ti}$s (pink spots) as a function of measured gap size.

Fig. S15

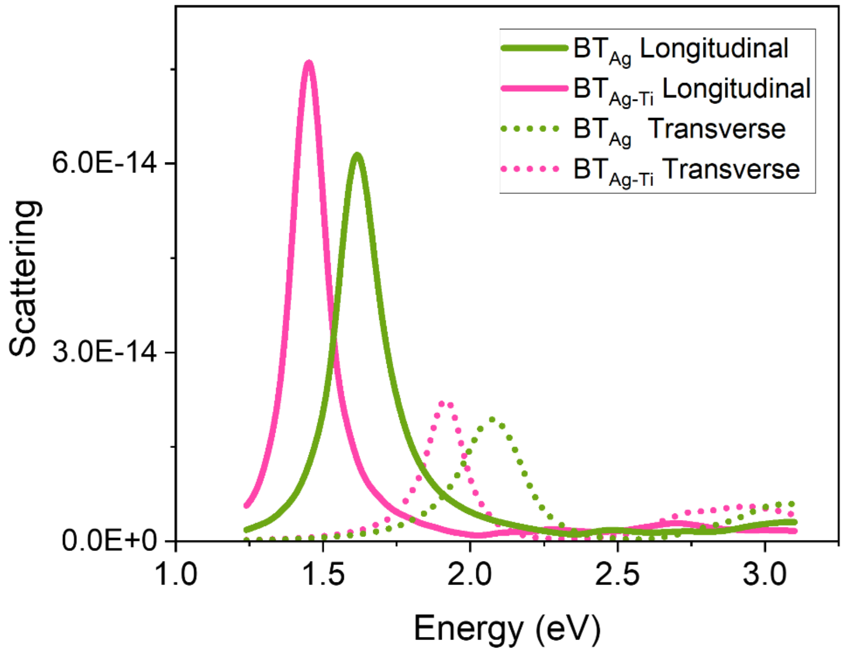

Figure S15. Numerical modeling of bowtie nanoantennas. Scattering spectra calculated using FDTD simulations for the longitudinal mode (solid green line) and transverse mode (dotted green line) of BT_{Ag}, and for the longitudinal mode (solid pink line) and transverse mode (dotted pink line) of $BT_{Ag–Ti}$. The prism length is 100 nm, the gap size is 20 nm, and the tip angle is 60°. The resonance energy, linewidth, and scattering intensity (defined as the area under the curve) of the longitudinal and transverse modes of BT_{Ag} are (1.62 eV, 177 meV, 1.70×10^{-14}) and (2.07 eV, 201 meV, 6.16×10^{-15}), respectively. The corresponding values for the longitudinal and transverse modes of $BT_{Ag–Ti}$ are (1.45 eV, 136 meV, 1.59×10^{-14}) and (1.91 eV, 123 meV, 4.24×10^{-15}), respectively.

Fig. S16

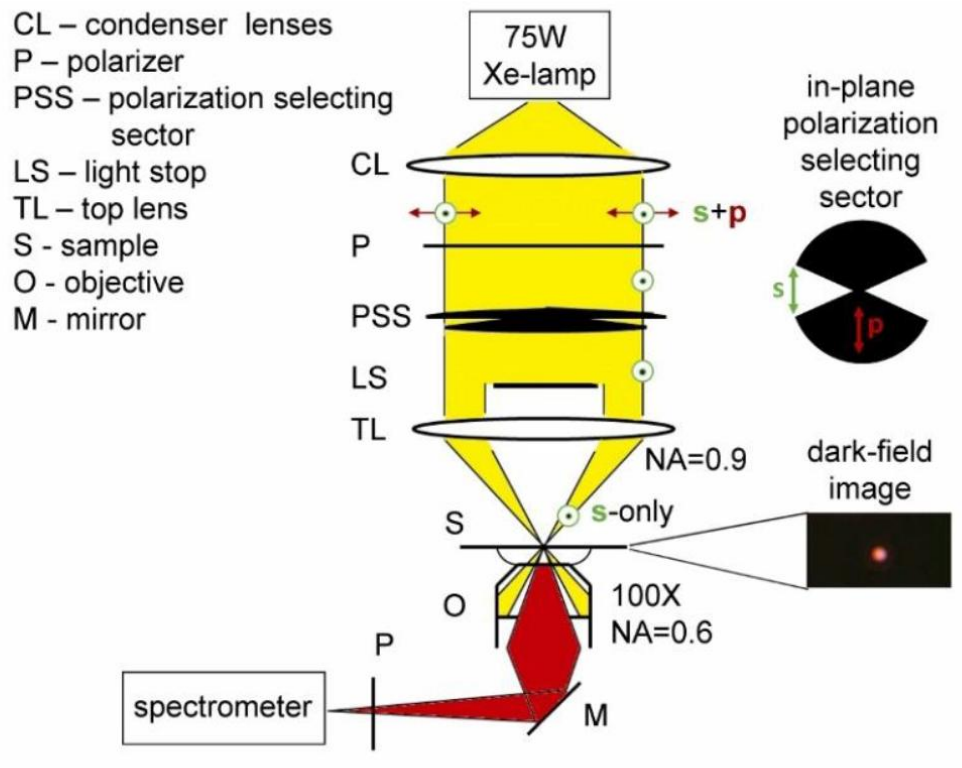

Figure S16: Schematic illustration of the optical setup used for dark-field scattering measurements. The system is based on an inverted microscope equipped with a dark-field condenser (NA = 0.9) and illuminated by a 75 W xenon lamp (Olympus). A combination of a light-stop and the upper condenser lens blocks normally incident light, ensuring illumination reaches the sample at an angle of approximately 70°. Scattered light is collected using a 100× oil-immersion objective and directed to a SpectraPro-150 spectrograph (Acton) equipped with a 1200 g/mm grating and a Newton CCD camera (Andor Technology). Excitation polarization is controlled using a polarizer together with a sector-shaped light-stop that restricts illumination to a narrow angular segment. When the polarizer transmission axis is oriented perpendicular to the bisecting line of the sector, the illumination at the sample is predominantly s-polarized. The setup configuration defines the excitation geometry, collection pathway, and spectral acquisition conditions used throughout all dark-field scattering experiments. Figure adapted from Ref. 10, originally published under the Creative Commons Attribution 4.0 International License (CC BY 4.0, http://creativecommons.org/licenses/by/4.0/).

Table S1

Table S1. Averaged linewidths, quality factors, resonance frequencies, and their standard deviations for the different experimental configurations.

Physical property measured	Mean (meV)	SD (meV)
Longitudinal linewidth in All BT_{Ag}	259.51	39.01
Longitudinal linewidth in All $BT_{Ag\text{-}Ti}$	206.03	34.2
Longitudinal Quality factor in All BT_{Ag}	7.75515	1.06351
Longitudinal Quality factor in All $BT_{Ag\text{-}Ti}$	9.75236	1.48209
Longitudinal linewidth in $BT_{Ag\text{-}Ti}$ without Al_2O_3	298.0714	41.5312
Longitudinal Quality factor in $BT_{Ag\text{-}Ti}$ without Al_2O_3	6.84676	0.88244
Transverse linewidth in All BT_{Ag}	252.9375	36.39683
Transverse linewidth in all $BT_{Ag\text{-}Ti}$	238.4667	30.87502
Transverse Quality factor in all $BT_{Ag\text{-}Ti}$	9.39079	1.44786
Transverse linewidth in $BT_{Ag\text{-}Ti}$ without Al_2O_3	261.2	76.36433
Transverse Quality factor in $BT_{Ag\text{-}Ti}$ without Al_2O_3	10.25343	3.14063
Longitudinal linewidth in BT_{Ag} with ω_0 =1.94 ± 0.02eV	257.2727	14.24142
Longitudinal linewidth in $BT_{Ag\text{-}Ti}$ with ω_0 =1.94 ± 0.02eV	189.8333	22.50993
Linewidth of all $Prism_{Ag}$s	233.47	35.18
Linewidth of all $Prism_{Ag\text{-}Ti}$s	221.84	28.66
Linewidth of $Prism_{Ag}$s with ω_0 =2.05 ± 0.05eV	213.4286	39.36097
Linewidth of $Prism_{Ag\text{-}Ti}$s with ω_0 =2.05 ± 0.05eV	216.8571	23.6476
Linewidth of all $BT_{Ti\text{-}Ag\text{-}Ti}$s, 0.2 nm Ti	197.3333	22.43615
Linewidth of all $BT_{Ti\text{-}Ag\text{-}Ti}$s, 1 nm Ti	203.4286	15.09809

Linewidth of all $BT_{Ti\text{-}Ag}$s	234.08	39.25655
ω_0 of longitudinal mode in all BT_{Ag}s	1.97436	0.08573
ω_0 of longitudinal mode in all $BT_{Ag\text{-}Ti}$s	1.99312	0.07551
ω_0 of transverse mode in all BT_{Ag}s	2.32115	0.1185
ω_0 of transverse mode in all $BT_{Ag\text{-}Ti}$s	2.20931	0.09643
ω_0 of all $prism_{Ag}$s	2.0497	0.17331
ω_0 of of all $prism_{Ag\text{-}Ti}$s	2.0155	0.09755
ω_0 of of all $BT_{Ti\text{-}Ag\text{-}Ti}$s, 0.2 nm Ti	1.98333	0.0574
ω_0 of of all $BT_{Ti\text{-}Ag\text{-}Ti}$s, 1 nm Ti	2.00857	0.06466
ω_0 of all $BT_{Ti\text{-}Ag}$s	1.922	0.09156

Table S2

Table S2. Mean and standard deviation of scattering intensity and normalized scattering intensity for BT_{Ag} and $BT_{Ag–Ti}$ across the three experiments shown in Fig. S12.

Physical property measured	Mean	SD	Normalized data Mean (a.u.)	Normalized data SD (a.u.)
Scattering intensity of BT_{Ag} (Fig. S12, panel a)	1.88E-25	3.88E-26	0.79367	0.16354
Scattering intensity of BT_{Ag-Ti} (Fig. S12, panel a)	1.13E-25	3.53E-26	0.47787	0.14906
Scattering intensity of BT_{Ag} (Fig. S12, panel b)	3.03E-25	8.87E-26	0.60139	0.17633
Scattering intensity of BT_{Ag-Ti} (Fig. S12, panel b)	1.43E-25	3.38E-26	0.28467	0.0672
Scattering intensity of BT_{Ag} (Fig. S12, panel c)	2.78E-25	7.49E-26	0.68373	0.18404
Scattering intensity of BT_{Ag-Ti} (Fig. S12, panel c)	1.47E-25	4.52E-26	0.36218	0.11107
